# Polysaccharide utilization loci-driven enzyme discovery reveals BD-FAE: a bifunctional feruloyl and acetyl xylan esterase active on complex natural xylans

**DOI:** 10.1186/s13068-021-01976-0

**Published:** 2021-05-31

**Authors:** Lisanne Hameleers, Leena Penttinen, Martina Ikonen, Léa Jaillot, Régis Fauré, Nicolas Terrapon, Peter J. Deuss, Nina Hakulinen, Emma R. Master, Edita Jurak

**Affiliations:** 1 Department of Bioproduct Engineering, University of Groningen, Nijenborgh 4, 9747 AG Groningen, The Netherlands; 2 Department of Bioproducts and Biosystems, Aalto University, Kemistintie 1, 02150 Espoo, Finland; 3 Architecture Et Fonction Des Macromolécules Biologiques (AFMB), UMR7257 Centre National de La Recherche Scientifique (CNRS) and Aix-Marseille Université (AMU), USC1408 Institut National de Recherche Pour L’Agriculture, l’Alimentation Et L’Environnement (INRAE), 13288 Marseille cedex 9, France; 4 Toulouse Biotechnology Institute (TBI), Université de Toulouse, CNRS, INRAE, INSA, Toulouse, France; 5 Department of Chemical Engineering, University of Groningen, Nijenborgh 4, 9747 AG Groningen, The Netherlands; 6 Department of Chemistry, University of Eastern Finland, Yliopistokatu 7, 80130 Joensuu, Finland; 7 Department of Chemical Engineering and Applied Chemistry, University of Toronto, 200 College Street, Toronto, ON M5S 3E5 Canada

**Keywords:** Feruloyl esterase (FAE), Acetyl xylan esterase (AcXE), Carbohydrate esterase (CE), Protein of unknown function (PUF), Polysaccharide utilization loci (PULs), Xylan, Enzyme discovery, Carbohydrate active enzymes (CAZymes)

## Abstract

**Background:**

Nowadays there is a strong trend towards a circular economy using lignocellulosic biowaste for the production of biofuels and other bio-based products. The use of enzymes at several stages of the production process (e.g., saccharification) can offer a sustainable route due to avoidance of harsh chemicals and high temperatures. For novel enzyme discovery, physically linked gene clusters targeting carbohydrate degradation in bacteria, polysaccharide utilization loci (PULs), are recognized ‘treasure troves’ in the era of exponentially growing numbers of sequenced genomes.

**Results:**

We determined the biochemical properties and structure of a protein of unknown function (PUF) encoded within PULs of metagenomes from beaver droppings and moose rumen enriched on poplar hydrolysate. The corresponding novel bifunctional carbohydrate esterase (CE), now named BD-FAE, displayed feruloyl esterase (FAE) and acetyl esterase activity on simple, synthetic substrates. Whereas acetyl xylan esterase (AcXE) activity was detected on acetylated glucuronoxylan from birchwood, only FAE activity was observed on acetylated and feruloylated xylooligosaccharides from corn fiber. The genomic contexts of 200 homologs of BD-FAE revealed that the 33 closest homologs appear in PULs likely involved in xylan breakdown, while the more distant homologs were found either in alginate-targeting PULs or else outside PUL contexts. Although the BD-FAE structure adopts a typical α/β-hydrolase fold with a catalytic triad (Ser-Asp-His), it is distinct from other biochemically characterized CEs.

**Conclusions:**

The bifunctional CE, BD-FAE, represents a new candidate for biomass processing given its capacity to remove ferulic acid and acetic acid from natural corn and birchwood xylan substrates, respectively. Its detailed biochemical characterization and solved crystal structure add to the toolbox of enzymes for biomass valorization as well as structural information to inform the classification of new CEs.

**Supplementary Information:**

The online version contains supplementary material available at 10.1186/s13068-021-01976-0.

## Background

Xylan is the most abundant hemicellulose and an important plant-derived polysaccharide for the production of bio-based chemicals, including xylitol, prebiotics, biofuels, and pharmaceuticals [1–5]. Its backbone consists of β-1,4-linked d-xylopyranosyl (Xyl*p*) residues which can be further substituted to varying degrees. The type and degree of substitution depends on the plant origin, e.g., cereals, grasses, softwood, or hardwood [6–8] and plant fraction, e.g., corn stover, corn cobs or corn bran [9, 10]. The most common substituents are α-1,2-(4-*O*-methyl)-d-glucuronic acids (MeGlc*p*A), α-l-1,2- and/or α-1,3-l-arabinofuranosyl (Ara*f*), and 2-*O*- and/or 3-*O-*acetyl. Moreover, Ara*f* can be further substituted with a 5-*O* linked feruloyl residue (Fa-Ara*f*) or with a complex oligomeric side chain [11]. For most applications, such as fermentation or chemical conversion, xylan requires degradation into smaller oligosaccharides or monosaccharides [1–4, 12, 13]. Enzymatic routes to xylan deconstruction employ Carbohydrate Active enZymes (CAZymes), which are classified into protein domain families by the CAZy database; www.cazy.org [14]. Glycoside hydrolases (GH) like e*ndo*-β-1,4-xylanases (GH5, GH10, GH11, GH30, GH98), xylobiohydrolases (GH30), *exo*-xylanases (GH8), β-xylosidases (GH3, GH39, GH43, GH52), α-xylosidases (GH31), α-l-arabinofuranosidases (GH43, GH51, GH62), α-glucuronidases (GH43, GH67, GH115) and α-l-galactosidases (GH95) cleave glycosidic bonds in the xylan backbone or of substituted sugars like Ara*f,* MeGlc*p*A and galactopyranoside [3, 15]. Carbohydrate esterases (CE) like acetyl xylan esterases (AcXE, CE1-7, CE16 [16–19]) and feruloyl esterases (FAE, distantly related to CE1 [20–24]) in turn release the ester-bound substituents acetic acid and ferulic acid. Complex substituents like the mentioned oligomeric side chains, however, hamper complete enzymatic saccharification [11, 25, 26]. The remaining recalcitrant oligosaccharides inform of the catalytic activities lacking in industrially used enzyme cocktails and provide suitable targets for discovery of such complementary functions within microbial genomes. Guilt-by-association is one approach to enzyme discovery, which exploits metagenomic information of lignocellulose-active microbial communities [27–30]. For example, searching polysaccharide utilization loci (PULs) (www.cazy.org/PULDB/) [31] for proteins of unknown function (PUFs), also referred to as unknowns (UNKs) or hypothetical proteins [32–34], has revealed novel enzymes acting on pectin [35], xylan [36, 37], galactomannan [38], chitin [39] and β-glucans [15, 28, 40–42]. PULs are physically linked gene clusters encoding CAZymes, carbohydrate binding modules (CBMs), carbohydrate transporters and PUFs that are simultaneously upregulated in the presence of a specific substrate to allow the synergistic degradation of target substrates [43–45]. They are encoded by the ubiquitous and abundant phylum of Bacteroidetes, whose species can harbor arsenals of more than a hundred PULs to tackle the wide glycan diversity [43].

Typically, 30–40% of predicted proteins are PUFs, and many are present in predicted PULs (www.cazy.org/PULDB/) [31, 34, 46]. PUFs can contain conserved Pfam domains [47], which are frequently not assigned to any function (Domains of Unknown Function) or assigned to large Pfam superfamilies in which the fold is conserved but functions can be highly diverse (e.g., α/β-hydrolases). In this study, we recombinantly produced, purified and characterized BD-FAE, a former PUF encoded within a PUL (BD-PH_PUL30) predicted to target xylan and originating from the metagenomes of beaver droppings and moose rumen enriched on poplar hydrolysate [27]. BD-FAE revealed either FAE or AcXE activity on various feruloylated and acetylated xylans. Phylogenetic and comparative genomic studies showed that its closest homologs appear in similar genomic contexts with xylan-degrading CAZymes clustering in PULs. Finally, the BD-FAE crystal structure was solved and co-crystallized to obtain a better understanding of the bifunctionality of this unclassified CE.

## Results

### Candidate selection and sequence analysis

**Fig. 1:**
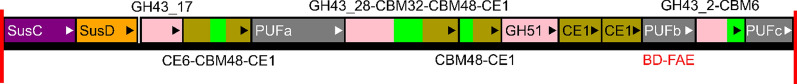
Schematic of BD-PH_PUL30 from a beaver gut metagenome and predicted catalytic activities. *SusC* Ton-B dependent outer membrane transporter (purple), *SusD* outer membrane binding protein (orange), *GH* glycoside hydrolase (pink), *CE* carbohydrate esterase (brown), *CBM* carbohydrate binding module (green) and *PUFa-c* Protein of unknown function (grey, named unknown (UNK) in PULDB, www.cazy.org/PULDB/ [31]), red bars indicate the margins of assembled region

A total of 303 PULs encoded by previously reported metagenomes from beaver dropping and moose rumen were annotated to verify CAZy and Pfam domain predictions [27]. In an effort to identify new xylan-active enzymes, PULs that comprised at least five predicted proteins and at least two CAZymes from families GH10, GH11, GH43, GH51, or GH115, were subject to further investigation. Among the resulting 15 PULs predicted to act on xylan, 6 comprised identical sequences and organization (2 being shorter likely due to incomplete assembly), and were found in both metagenomes enriched on poplar hydrolysate (Fig. 1). The corresponding PUL encoded three PUFs; PUFb (subsequently named BD-FAE) encodes a predicted signal sequence for secretion and an α/β-hydrolase fold (PF12695) [47], which motivated its selection for functional characterization.

**Fig. 2:**
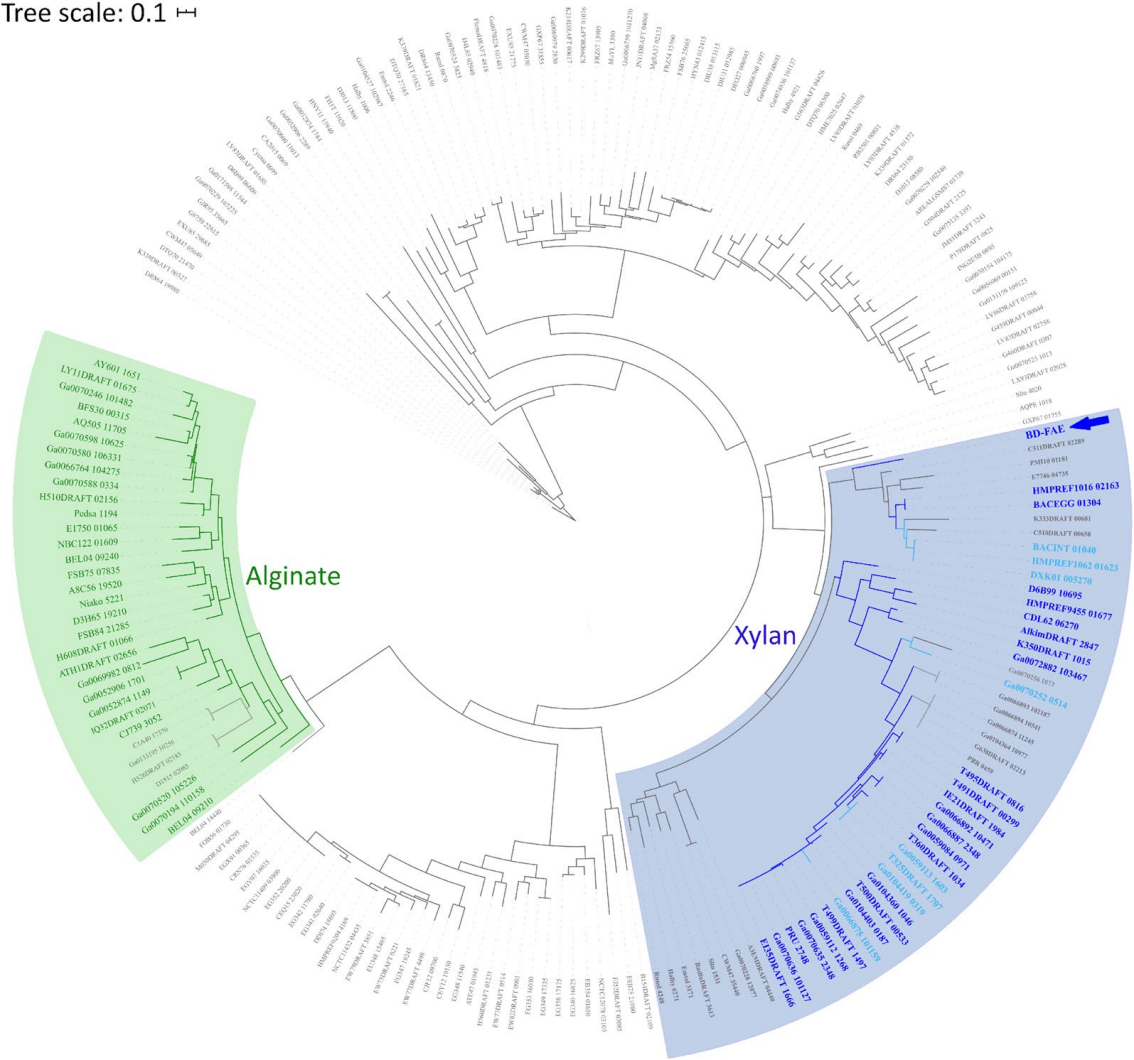
Phylogeny of BD-FAE homologs in PULDB. The blue background highlights the monophyletic clade gathering BD-FAE with its homologs predicted in a xylan PULs (leaf label in a bold-blue font; dark = high confidence; light = putative). The green background highlights the monophyletic clade gathering all homologs appearing in an alginate PUL (leaf label in bold-green font)

BD-FAE comprises parts of two Pfam domains, namely Abhydrolase_3 and Peptidase_S9 (PF07859 and PF00326, respectively). More precisely the N-terminal sequence matches the first half of PF07859 family model, while the C-terminal sequence matches the second half of PF00326 family model. Unlike other described FAEs, BD-FAE does not display the remote homology to CE1 family, and likely belongs to a novel broad esterase family to be created in dedicated databases (e.g., ESTHER [48]). Sequence similarity search against the non-redundant NCBI database [49] revealed that BD-FAE homologs mostly belong to the Bacteroidetes phylum and to the α/β-hydrolase superfamily. Given this taxonomic specificity, a sequence similarity search was conducted against the 1,283 Bacteroidetes genomes integrated in PULDB (www.cazy.org/PULDB/ [31], accessed on 14.06.2020) revealing 200 homologs separated into two groups (Additional file 1: Table S1). Group 1 contained 53 homologs, of which 33 were encoded in PULs predicted to act on xylan. The majority of homologs in Group 2 are not encoded in PULs; however, 29 were identified in alginate-targeting PULs. A phylogenetic analysis of all 200 homologs showed BD-FAE as the basis of a monophyletic clade gathering all BD-FAE homologs identified in PULs predicted to act on xylan, and clearly separated from homologs not associated with PULs or else in PULs predicted to act on alginate (Fig. 2).

### Enzyme production and initial activity screen

BD-FAE and a truncated form (ΔMet1-Pro7) were successfully expressed in *E. coli* BL21 (DE3) and purified as soluble protein with yields of 18 mg/L and 16 mg/L, respectively, and with high purity (Additional file 2: Figures S1A, S2A). Their respective molecular mass was 32,511 Da and 31,633 Da and corresponded to those calculated from the primary sequence (ProtParam server [50]). The oligomerization states of both proteins in solution were examined by native mass spectrometry, dynamic light scattering, and size exclusion chromatography, which revealed both proteins existed mainly as monomers and dimers in solution with minor indication of higher oligomers (Additional file 2: Figure S2).

The catalytic activity of BD-FAE was first assessed in an initial screening on 9 *p*NP-glycosides, 1 *p*NP-ester, and 17 polysaccharides (Additional file 2: Table S2) at 3 pH values (5.5, 7.0, 8.5) and 3 time points (2 h, 4 h, 24 h). Acetyl esterase activity was detected on *p*NP-acetate (*p*NP-Ac) between pH 5.5 and pH 7.0 (Additional file 2: Figure S3), while no hydrolytic activity was detected on any of the 17 polymeric substrates tested in the initial screening (Additional file 2: Table S2).

### Biochemical characterization using synthetic substrates

**Fig. 3:**
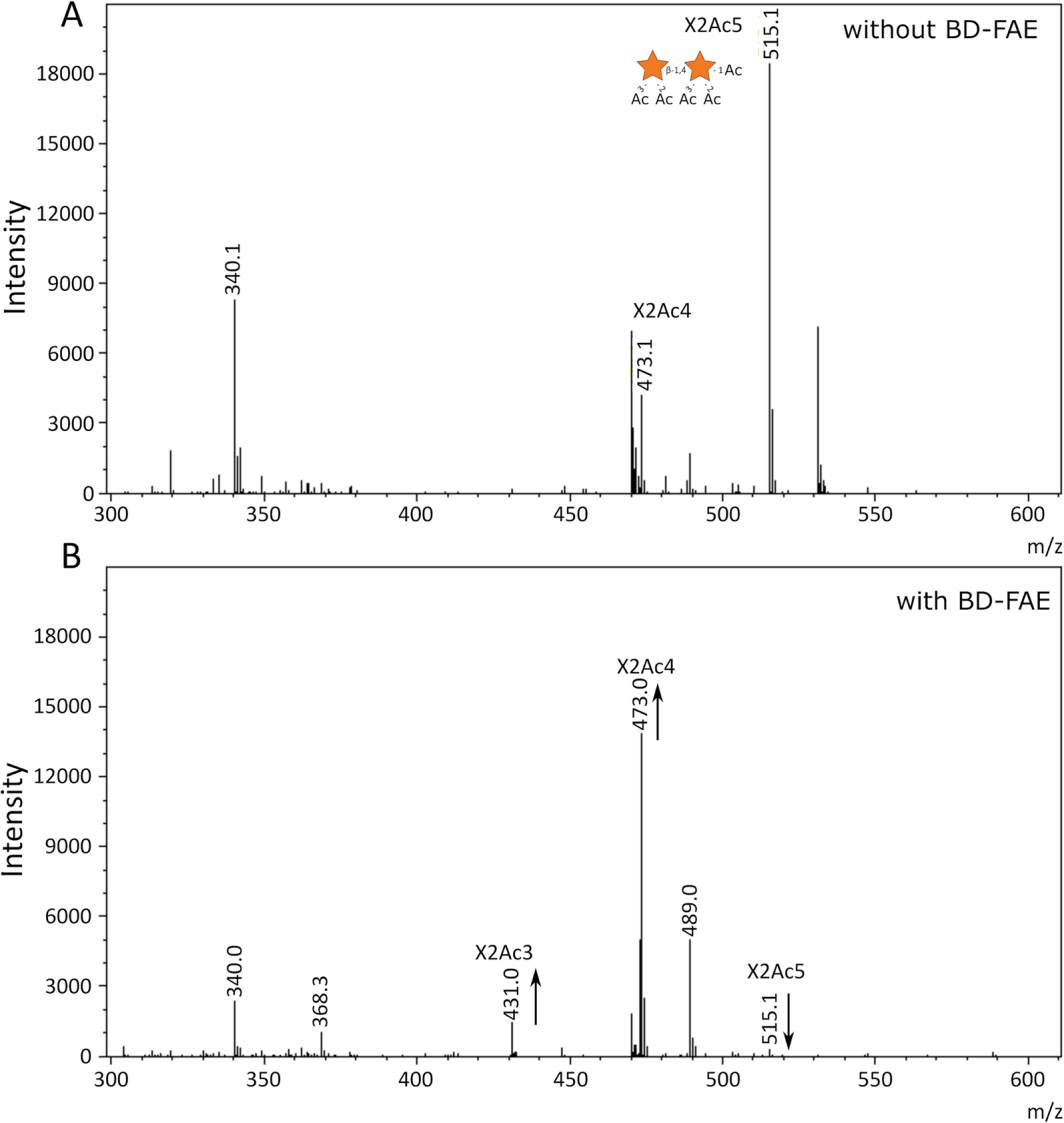
MALDI-TOF spectra before (**A**) and after (**B**) incubating 3% (g enzyme / g dry matter substrate) BD-FAE on X2Ac5 (2,3-di-*O*-acetyl-β-d-Xyl*p*-(1,4)-1,2,3-tri-*O*-acetyl-α-d-Xyl*p*) (1 mg/mL final concentration) at pH 7.0 and 40 °C for 24 h. All m/z values are sodium adducts. X = orange star = xylosyl, Ac = acetyl residues (*n* = 2)

**Fig. 4:**
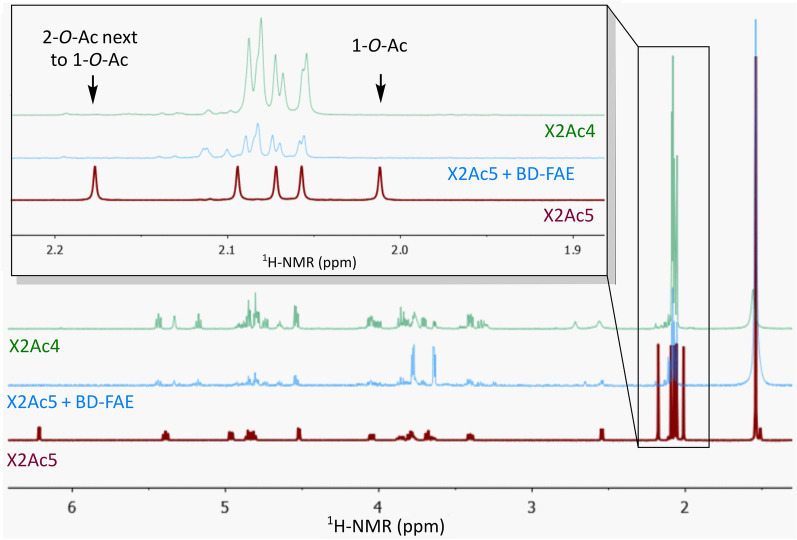
Comparison of the ^1^H-NMR spectra of X2Ac5 and X2Ac4 substrate blanks in CDCl_3_ and the product of X2Ac5 after treatment with BD-FAE extracted into CDCl_3_ showing the disappearance of the acetyl signal corresponding to 1-*O*-Ac and formation of a signal pattern corresponding to X2Ac4 (where the signal of 2-*O*-Ac shifts to overlap with the other acetyl signals)

1-Naphthyl acetate was used to evaluate the pH optimum of BD-FAE, which was determined to be between pH 6.0 and pH 7.0 (Additional file 2: Figure S1B). The kinetic parameters of BD-FAE on *p*NP-Ac (*K*_m_ of 2.29 ± 0.03 mM, and *k*_cat_ of 0.89/s; Additional file 2: Figure S1C) revealed low catalytic activity compared to characterized acetyl esterases on the same substrate (Additional file 2: Table S3) [37, 51–53]. Moreover, substrate inhibition for BD-FAE was observed with a *K*_i_ of 14 ± 5 mM *p*NP-Ac. AcXE activity and positional specificity were therefore evaluated using more complex synthetic substrates, namely two acetylated xylobioses (X2Ac5: 2,3-di-*O*-acetyl-β-d-Xyl*p*-(1,4)-1,2,3-tri*-O*-acetyl-α-d-Xyl*p* and X2Ac4: 2,3-di*-O*-acetyl-β-d-Xyl*p*-(1,4)-2,3-di-*O*-acetyl-d-Xyl*p*). After a 4-h incubation, BD-FAE had released 18% of the total acetic acid from X2Ac5 and 13% from X2Ac4. After 24 h, X2Ac5 was almost entirely converted to X2Ac4 and X2Ac3 (Fig. 3), and X2Ac4 was partially converted to X2Ac3 (Additional file 2: Figure S4). The positional specificity of BD-FAE was further analyzed by ^1^H-NMR, which showed preference towards the 1-*O*-Ac position of the synthetic substrate X2Ac5 (Fig. 4).

**Fig. 5:**
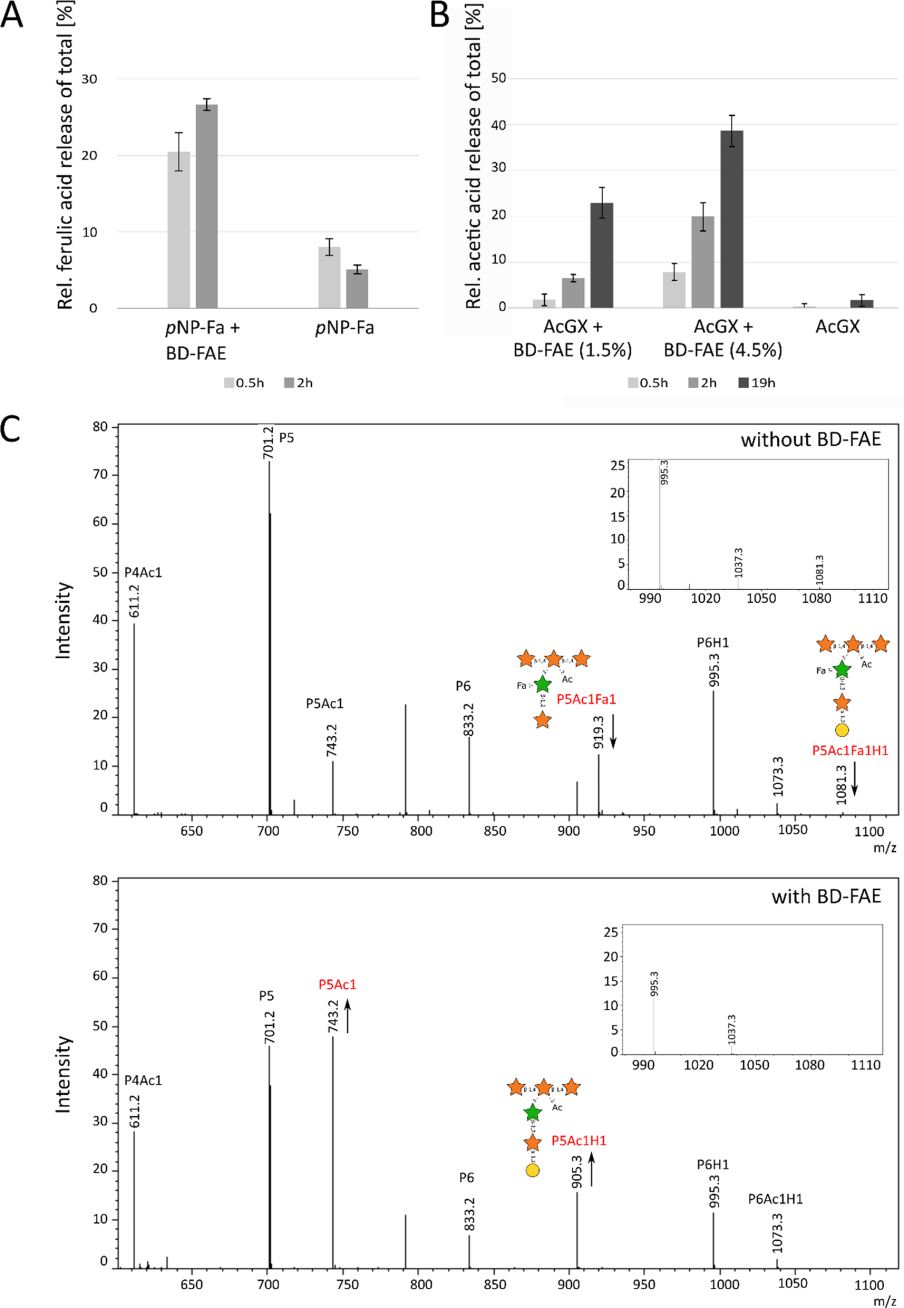
Carbohydrate esterase (CE) activity of BD-FAE. **A** 1 µg BD-FAE on 1 mM *p*NP-ferulate at pH 7 and 40 °C, n = 2. **B** 1.5% and 4.5% (g enzyme / g dry matter substrate) BD-FAE on acetylated glucuronoxylan (AcGX, 15 mg/mL final concentration) at pH 6.0 and 40 °C analyzed with acetic acid kit (K-ACETRM, enzyme and buffer blanks subtracted, see Additional file 2: Table S4), *n* = 2 and **C** MALDI-TOF spectra before and after incubating 3% (g enzyme / g dry matter substrate) BD-FAE on 10 mg/mL acetylated and feruloylated xylooligosaccharides from corn fiber (AcFaXOS) at pH 7.0 and 40 °C for 4 h (*n* = 2). All m/z were sodium adducts. Structural annotation is based on [11]. *P* pentose, *H* hexose, *Ac* acetyl, *Fa* feruloyl, *orange star* d-xylosyl, *green star* l-arabinosyl, *yellow circle* l-galactosyl residues

To investigate whether BD-FAE can accept larger ester-bound ligands, the enzyme was tested for glucuronoyl esterase (GE) and feruloyl esterase (FAE) activity using benzyl-d-glucuronate (BnGlcA) and *p*NP-ferulate (*p*NP-Fa), respectively. GE and FAE activities are especially relevant to the breakdown of lignin–carbohydrate complexes that connect hemicelluloses and lignin [21, 54–56]. While no GE activity was detected (Additional file 2: Figure S5), BD-FAE released 22% of the total ferulic acid of *p*NP-Fa after 2 h (Fig. 5A, substrate blank subtracted).

### Biochemical characterization using natural substrates

BD-FAE released over 20% of the total acetic acid from acetylated glucuronoxylan (AcGX) after 2 h and over 35% after 19 h (Fig. 5B). No acetic acid release from acetylated galactoglucomannan (AcGGM) was observed. The lack of acetyl esterase activity towards the mannan-based substrate suggests a preference towards xylans, which was consistent with the predicted substrate specificity of BD-PH_PUL30. Further investigation of FAE activity was carried out by incubating BD-FAE on highly substituted xylooligosaccharides from corn fiber (AcFaXOS), which were previously classified as recalcitrant towards industrial pre-treatment methods by Appeldoorn and co-workers [11]. Next to 2-*O*-Ac or 3-*O*-Ac single substitutions some Xyl*p* units of the xylan backbone were decorated with both 2-*O*-Ac and an oligomeric side chain consisting of α-l-galactopyranosyl-(1,2)-β-d-Xyl*p*-(1,2)-[5-*O-trans*-feruloyl]-l-ra*f* (Fig. 5C). Interestingly, BD-FAE was capable of completely removing feruloyl residues of these AcFaXOS (m/z 1081.3 and 919.3), but the acetyl substituents remained untouched (Fig. 5C).

### Crystal structures and substrate binding of BD-FAE and its truncated form

**Fig. 6:**
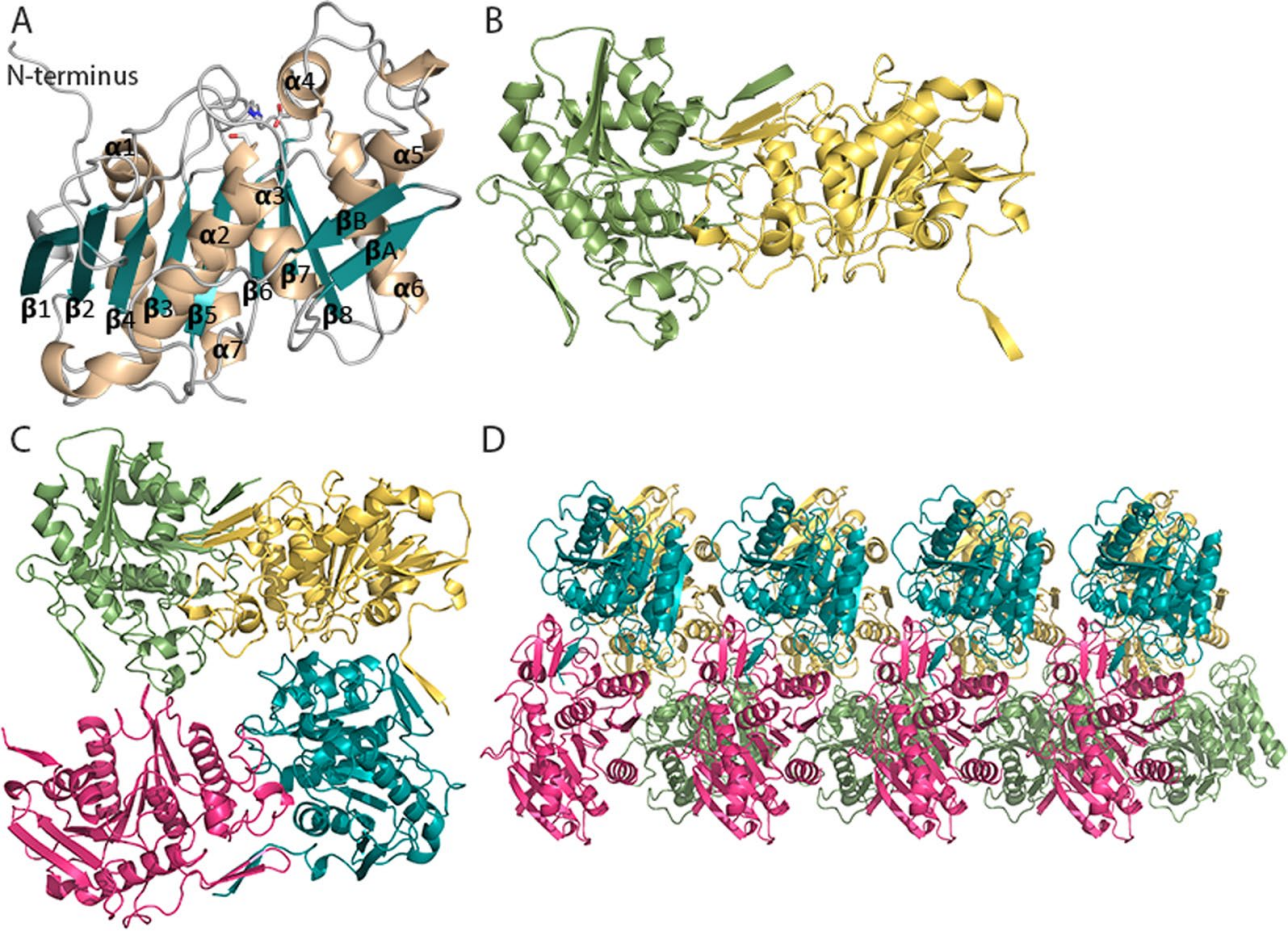
The crystal structure of BD-FAE. **A** α-helices are shown in brown and β-sheets in turquoise. Catalytic residues Ser128, Asp237 and His269 are marked in sticks. **B** In the crystal, the N-terminal tail of one BD-FAE molecule packed against the small β-sheet on the surface of an adjacent BD-FAE molecule in a consecutive manner. **C** In front view it looks like a tetramer **D** but in side view it can be seen that the packing formed a fourfold spiral shaped polymer

**Fig. 7:**
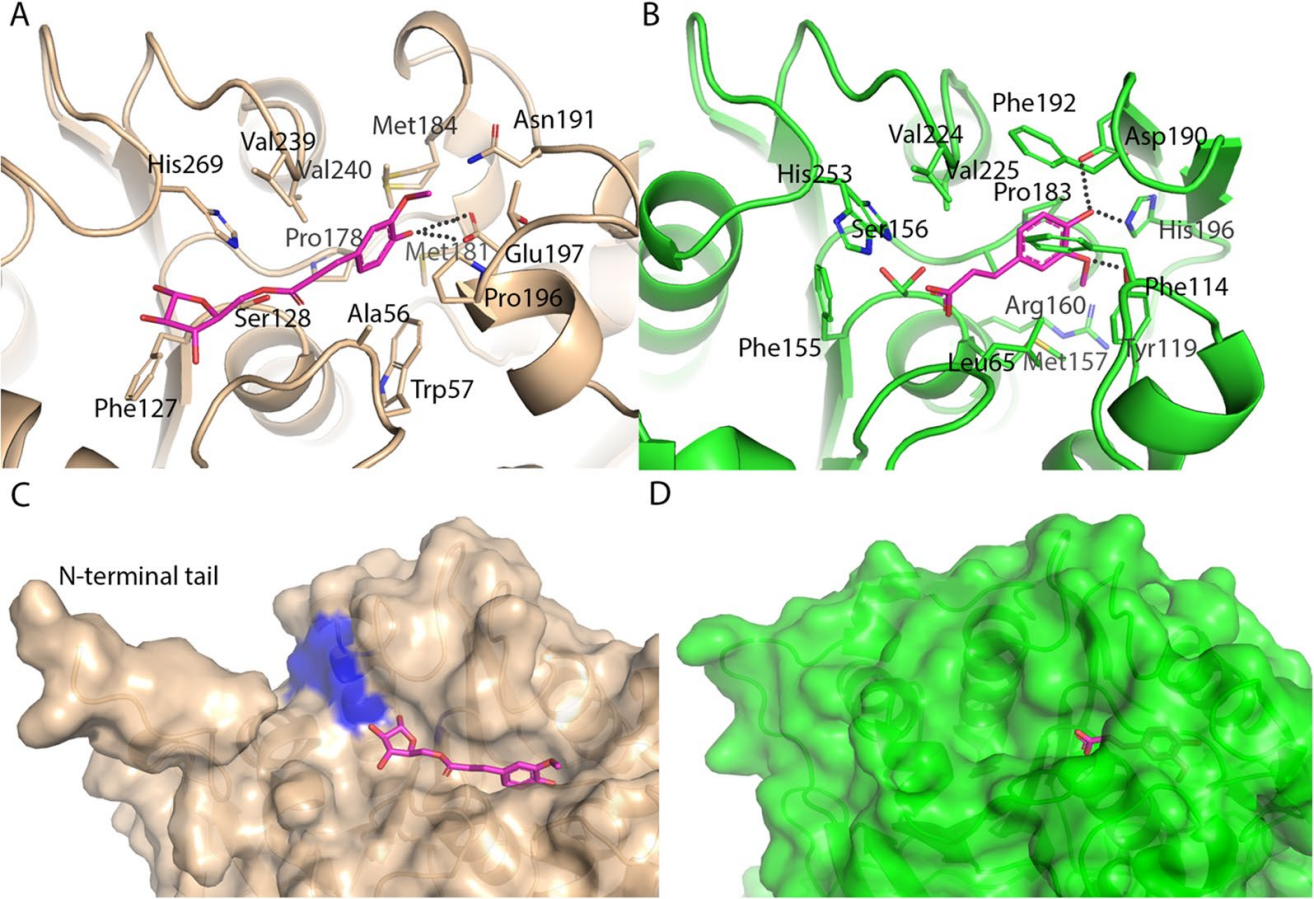
Comparison of substrate binding in BD-FAE (beige) and FAE from *Anaeromyces mucronatus* (*Am*CE1, PDB: 5CXX, green). The amino acid residues that were near the ligand were shown as sticks. The ligand was shown in pink and the hydrogen bonds of it were shown as dotted lines. **A** Fa-Ara*f* docked into BD-FAE’s active site. **B** Complex structure of *Am*CE1a with ferulic acid. Surface representations of **C** BD-FAE and **D** *Am*CE1 showed that the active site of BD-FAE was more solvent exposed, whereas in *Am*CE1, the active site was more pocket like. Aliphatic residues on the surface of BD-FAE form a possible xylan-binding cleft, marked in blue

The crystal structure of BD-FAE was solved (PDB: 6TKX) to predict structural determinants that likely drive AcXE and FAE activities of the enzyme. The BD-FAE structure belonged to space group P4_3_2_1_2 and contained one molecule in an asymmetric unit. The final model of BD-FAE was refined to 2.06 Å resolution and it contained the residues from Gln2 to Glu292 (Additional file 3: Table S5). Clear electron density permitted unambiguous modeling of all residues except Met1, Leu293 and Glu294. The His-tag was not visible and the N-terminal tail showed weaker electron density than other residues of the protein, most likely due to its flexible protruding nature. Overall, the BD-FAE crystal structure adopted a typical α/β-hydrolase fold (Fig. 6), which was consistent with the above mentioned BLASTp results. The central β-sheet consisted of eight β-strands, named β1-β8 (Fig. 6A). Seven of them were aligned in parallel fashion while β2 was aligned in anti-parallel fashion. The central β-sheet was surrounded by seven α-helices (α1-α7), which together formed the α/β/α-core-structure. A second small anti-parallel β-sheet, also called β-hairpin, consisted of the two β-strands (βA and βB) formed by the residues Thr145-Asp154 after β5-strand and α3-helix. It was located opposite of the active center. The active center contained the conserved catalytic triad of Ser128, Asp237 and His269 (Fig. 7A) and the oxyanion hole was composed of NH-groups of Gly53 and Ser128. Looking at the surface model it can be seen that the active site was solvent exposed and formed a shallow furrow (Fig. 7C). A comparison with other solved CE crystal structures can be found in the Additional file 3: Figure S7 with GH43-CE of *Bacteroides eggerthii* (PDB: 6MLY) being the closest hit sharing only 55% sequence identity.

The solved BD-FAE structure revealed higher-order oligomers within the corresponding crystal (Fig. 6B–D). The N-terminal tail (Gln2-Pro7) protruded out of the core protein and packed against an adjacent symmetry molecule in the crystal (Fig. 6A, B). Such crystal packing did not form a closed-ended dimer but an unusual fourfold spiral shaped polymer, in which the active sites pointed to the center of the spiral (Fig. 6C, D). This open-ended oligomerization could be described as fibril formation [57]. The interaction area of sequential molecules within the polymer was determined using PISA server [58] and revealed 1055 Å^2^, which was typical for strong intermolecular interactions. To investigate the role of the first seven N-terminal residues in oligomerization, the crystal structure of a truncated form (ΔMet1-Pro7, PDB: 6XYC) was solved. The final model of the truncated form was refined to 1.85 Å resolution and contained residues from Met8 to Lys292 (Additional file 3: Table S5). Surprisingly, also in the crystal of the truncated form, higher-order oligomers were observed indicating that N-terminal deletion was not sufficient for disrupting this assembly.

Substrate binding in BD-FAE was investigated by crystal soaking followed by co-crystallization and docking studies. The first approach was used to obtain a complex structure with a bound XOS ligand (degree of polymerization (DP) 1–6), however, no electron density for any ligand was detected and no changes in loop orientation or oligomerization were found. Docking studies with XOS (DP 1, 2, 4, and 6) as ligands were performed to explore the possibility of substrate binding onto the interface of two neighboring BD-FAE molecules and thus whether the unusual oligomerization could play a functional role. The search space was set to the active site of BD-FAE, to the whole molecule or to two BD-FAE molecules packed equally to its oligomeric form in the crystal but no clear binding was observed. In the crystal structure of BD-FAE, a sulfate ion was bound to the active site, which originated most likely from the ammonium sulfate containing crystallization solution (Additional file 3: Figure S6A). It mimicked the binding of an acetyl group to the catalytic triad. In the crystal structure of the truncated form, the serine protease inhibitor AEBSF (4-(2-aminoethyl)benzenesulfonyl fluoride) of the lysis buffer covalently bound to the active site’s Ser128 (Additional file 3: Figure S6B). The phenolic ring in bound AEBS moiety resembled the binding of ferulic acid and tetrahedral sulfonyl of Ser-AEBS mimicked the enzyme–substrate–intermediate during carboxylic acid binding at Ser128. Based on that observed complex structure, Ara*f* substituted with a 5-*O* linked feruloyl residue (Fa-Ara*f),* a common substituent of xylans, was successfully docked into the active site of BD-FAE (Fig. 7A, C). The amino acid residues that bound Fa-Ara*f* in BD-FAE were similar to those binding the AEBS moiety (Additional file 3: Figure S6), and were in a long α-helical loop after the β5 strand. A stabilizing disulfide bond between Cys186 and Cys242 prevented extensive movement of that loop. The phenol of ferulic acid was sandwiched between Pro196 and Val240 by π-CH stacking and van der Waals interactions and the furanose ring of Ara*f* interacted with Phe127 via CH-π stacking (Fig. 7A). Overall, similarities in binding small substrates were observed to the characterized fungal FAE of *Anaeromyces mucronatus* (*Am*CE1/Fae1A, PDB: 5CXX [59, 60], Fig. 7)*.* The hydroxyl group of ferulic acid bound in BD-FAE to Glu197 in a bidentate way (Fig. 7A). In *Am*CE1, the corresponding residue was Asp190 and overall hydrogen bonding of ferulic acid with surrounding amino acid residues was stronger than in BD-FAE (Fig. 7B). The importance of Asp190 for substrate binding in *Am*CE1 was shown by mutating it to alanine, which led to a drastically decreased FAE activity [60]. Thus, Glu197 in BD-FAE likely plays a similar role as an important residue for FAE activity.

## Discussion

Few CE family members have been biochemically characterized and the number of available crystal structures is limited [19, 22, 61, 62]. The sequences of CEs with similar catalytic activities often show low identity, which has hampered sequence-based classification [18, 19, 22, 63]. Moreover, many sequence-based esterase families show low substrate specificity, frequently including members that act on substrates beyond carbohydrates [61]. At the same time, the tertiary structures of CEs typically adopt an α/β-hydrolase fold shared with serine proteases, peroxidases, lipases, epoxide hydrolases and dehalogenases [64–66] providing little, if any, identifying structural features for classification. Thus, it is not possible to predict confidently a catalytic function or a substrate specificity based only on sequence-based family or structural similarities. Therefore, a thorough biochemical characterization on natural substrates is indispensable to ensure correct classification of esterases into subfamilies with a reliable predictive power [18, 19, 22, 67].

### Phylogeny, genomic context and catalytic activity

Looking at the genomic context of BD-FAE, the presence of four putative α-l-arabinofuranosidases (GH43, GH51) and five predicted CEs (CE1, CE6) in BD-PH_PUL30 suggested catalytic activities capable of removing xylan-specific substitutions like Ara*f*, acetyl and feruloyl residues (Fig. 1). Such a substrate could be highly substituted arabinoxylan for example originating from cereals, as arabinoxylan is the most common xylan within the group of grasses (Poaceae). Prior to biochemical characterization, speculations on the different roles and putative synergistic capacities of the other encoded proteins in the PUL would be error-prone, especially due to the broad substrate range of CEs. Nevertheless, closest homologs of BD-FAE form an independent clade in which most members belong to xylan-targeting PULs, shaping a subfamily dedicated to xylan degradation. Together with the repeated occurrence of BD-PH_PUL30-like clusters in the metagenomic dataset [27], this supports the likelihood that BD-FAE and its homologs have an important function in microbial xylan degradation (Fig. 2). To ensure a nonbiased characterization, however, the initial screening of BD-FAE was performed on a broad substrate library covering 10 simple *p*NP-glycosides and *p*NP-Ac as well as 17 complex natural, polymeric substrates (Additional file 2: Table S2). Following detection of acetyl esterase activity on *p*NP-Ac in the initial screening and a rather low *k*_cat_ value of 0.89/s, catalytic activity of BD-FAE was further studied on other substrates with increasing complexity. Of the tested *p*NP-esters, *p*NP-Ac and *p*NP-Fa BD-FAE released 0.22 nmol *p*NP/ μg and 0.22 mmol *p*NP/ μg after 2 h, respectively, suggesting a preference for the feruloylated substrate (Additional file 2: Figure S3, Fig. 5B). On X2Ac5 positional specificity of BD-FAE towards 1-*O*-Ac was observed (Fig. 4), consistent with the degradation of X2Ac5 into X2Ac4 (Fig. 3). This linkage, however, does not occur in natural xylans [6, 8, 9] but might be the most accessible or reactive acetyl group in this synthetic substrate. We also showed that BD-FAE was capable of releasing 37% of total acetic acid from AcGX within 19 h (Fig. 5B) and 13% of total acetic acid from X2Ac4 after 4 h, in which the acetyl groups are linked 2-*O* and/or 3-*O* to a Xyl*p*. These results point out that catalytic activity and positional specificity can differ on synthetic and natural substrates [24, 68]. BD-FAE did not act on AcGGM containing 2-*O* and/or 3-*O* acetylated d-mannose units [69], which is in line with BD-FAE being encoded in a xylan-related PUL. Thus, BD-FAE was capable of removing acetyl residues from synthetic and natural xylan-based substrates. On highly substituted AcFaXOS from corn fiber BD-FAE completely removed the feruloyl substituents while the acetyl residues remained untouched on this substrate (Fig. 5C). The AcFaXOS are heavily substituted with 2-*O*-Ac or 3-*O*-Ac single substitutions and Xyl*p* units with 2-*O*-Ac substitutions can be further decorated with a bulky oligomeric side chain (Fig. 5C) [11, 25]. Therefore, even though BD-FAE partially deacetylated AcGX (Fig. 5B) and acetylated xylobioses (Fig. 3), the absence of detectable acetic acid release from highly substituted AcFaXOS could be explained by steric hindrance of the oligomeric side chain next to the *O*-2 bound acetyl group.

The overall catalytic activity of BD-FAE was comparable to type-A FAEs of Crepin’s classification system [70]. Members of this type are capable of removing ferulic acid from synthetic substrates and show lower catalytic activity towards acetylated substrates. Further, type-A FAEs show a strong preference for 5-*O*-Fa-α-l-Ara*f* present in xylans compared to 2-*O*-Fa-α-l-Ara*f,* which occurs in sugar beet pectin and in spinach [71, 72]. Thus, it was not expected that BD-FAE, which is encoded in a PUL suggested to target xylan and showing similarities to type-A FAEs, is capable of removing 2-*O*-Fa-α-l-Ara*f*. Finally, BD-FAE showed comparable catalytic activity to the recently characterized fungal bifunctional esterase FaeD from *Podospora anserina* S mat + [24]. Although not similar at the sequence level, both are capable of releasing acetic acid and ferulic acid from synthetic model substrates and more complex xylan-based substrates. For example, BD-FAE released 37% of total acetic acid from birchwood xylan after 19 h, whereas FaeD released 35% of total acetic acid from wheat-derived xylooligosaccharides after 24 h [24]. Moreover, both enzymes show higher relative activities towards feruloylated substrates as compared to acetylated substrates.

### Analyzing substrate binding in BD-FAE

The catalytic triad of Ser-Asp-His is conserved throughout AcXEs and FAEs. Thus, it is suggested that the surroundings of the active site play an important role in substrate specificity [60, 73–75]. The wide, solvent exposed active site of BD-FAE forms a shallow furrow that could sterically enable the binding of highly substituted bulky substrates (Fig. 7C). This is in line with the biochemical characterization of BD-FAE, revealing AcXE and FAE activity not only on simple synthetic substrates, but also on highly substituted xylans (Fig. 5B, C). The observation that BD-FAE can remove feruloyl residues from AcFaXOS but not the adjacent acetyl substituents suggests steric hindrance likely due to the complexity of the oligomeric side chain. The carbohydrate backbone of the substrate or 5,5ʹ-diferulates cross-linking, e.g., two chains of arabinoxylan might interact with aromatic residues that surround the active site via π-stacking interactions (Fig. 7C). To analyze how BD-FAE would bind a xylan chain several XOS were used to soak crystals followed by co-crystallization, or XOS were docked into the crystal structure. However, in the crystals no electron density for the ligands were found and docking displayed unspecific binding to the N-terminal tail. These results suggest that specific binding of a carbohydrate chain is not needed for successful catalytic activity as shown for *An*FaeA of *Aspergillus niger* [76] and consistent with BD-FAE activity on simple model substrates (Fig. 5B, C). Another explanation would be that for binding a xylan chain substituents are needed. Finally, BD-FAE’s binding cleft was compared to the fungal *Am*CE1 due to similarities in binding feruloyl residues (Fig. 7C, D; [59, 60]; 5CXX). The active site of *Am*CE1, however, is burrowed and no binding cleft for longer substrate chain is found, which was in line with its proposed specific exolytic FAE activity. The FAE activity of *Am*CE1 was only confirmed on methyl ferulate and thus it is unknown whether larger oligomeric substrates are accepted as substrates. Overall, it is notable that most FAEs were tested on small model substrates only [20, 21]. Therefore, it is unclear whether the ability to bind complex substituted substrates is a common feature of FAEs.

### The role of the N-terminal tail in oligomerization

In crystals of BD-FAE, the protruding N-terminal tail packed as a β-strand against a small β-sheet on the surface of another molecule, leading to an unusual fourfold spiral shaped polymer (Fig. 6). Surprisingly, the crystal structure of an N-terminal truncated form of BD-FAE (∆Met1-Pro7) adopted a similar spiral shaped polymer. The interaction surface area of sequential molecules in the truncated form, however, was determined to be 764 Å^2^, which is 28% smaller compared to BD-FAE (1055 Å^2^). N-terminal residues were previously shown to participate in protein packing; for example the N-terminal β-domains of two adjacent *Bi*Fae1A monomers, an FAE from *Bacteroides intestinalis,* lead to dimerization and subsequent tetramerization (PDB: 5VOL [73]). Moreover, open-ended polymers or filaments of protein have been discovered among metabolic enzymes such as cytidine triphosphate synthase, in which polymer formation regulates the amount of free enzyme in the cell [77]. Free enzymes were catalytically active, while enzymes packed into a polymer were inactive [77]. An activator molecule initiated dissociation of enzymes from a polymer, which in the case of BD-FAE could be the correlating substrate.

## Conclusion

BD-FAE, a previously unknown protein encoded in a metagenomic PUL from beaver droppings belongs to the functionally diverse α/β-hydrolase superfamily. We demonstrated that BD-FAE removes feruloyl and acetyl groups from simple model substrates, acetyl groups from birchwood glucuronoxylan, and feruloyl groups from highly substituted AcFaXOS from corn fiber. Thus, its family might display various substrate specificities across subfamilies. The solved BD-FAE crystal structure revealed a shallow furrow for substrate binding that could accommodate substituted bulky substrates. Together, our phylogenetic, biochemical, and structural analyses suggest that BD-FAE is the founding member of a new esterase family.

## Methods

### Candidate selection

Annotation of protein domains in the previously published metagenomic dataset [27] relied on HMMER searches [78] using Pfam [47] and CAZy library [14] with recommended thresholds. PUL prediction was performed similarly to the PULDB (www.cazy.org/PULDB/ [31]), with a relaxed procedure that only require *susD* presence to start, and not necessarily *susC,* to cope with the fragmentary aspect of metagenomic dataset. PULs containing more than five genes encoding CAZy family members related to xylan degradation (GH10, GH11, GH43, GH51, GH115 and CE1) as well as PUFs were further investigated. To verify the quality of the selected PUL (BD-PH PUL30, Fig. 1) each protein was analyzed for its sequence length (> 250 bp) and homologs (BLASTp [49]), the present of a signal peptide of Gram-negative bacteria (SignalP5.0 [79]), putative pfam domains [47], and their putative secondary structure (JPred 4 [80]). Based on these results, PUFb of BD-PH_PUL30, subsequently named BD-FAE, was selected for in-depth functional characterization.

### Phylogeny

A BLASTp [49] search against the NCBI non-redundant database was performed using BD-FAE as a query with a 10E-10 e-value threshold and resulted in 153 hits out of which only seven belonged to other phyla than the Bacteroidetes. Subsequently, a BLASTp search against all proteins encoded by the 1283 genomes integrated in the PULDB (www.cazy.org/PULDB/ [31], accessed on 14.06.2020) was performed. The first 200 hits were retrieved and further analyzed (Additional file 1: Table S1). Genomic contexts of the homologs were manually inspected using the genome browsers of the PULDB. Glycan structure targeted by PULs was determined based on known CAZymes specificities. To visualize the phylogenetic relationship and the genomic context of these 200 homologs of BD-FAE, a phylogenetic analysis was performed at ngphylogeny.fr with “A la carte” settings [81]. A multiple sequence alignment (MAFFT [82]) was created and cleaned with Block Mapping and Gathering using Entropy (BMGE [83]). The final tree was reconstructed with PhyML based on maximum-likelihood and visualized with interactive Tree of Life (iTOL [84]; Fig. 2).

### Substrates & chemicals

All *para-*nitrophenyl-bound (*p*NP-) and most polymeric substrates of the initial screening (Additional file 2: Table S2), acetylated glucuronoxylan (AcGX, birchwood), K-ACETRM kit and K-URONIC kit were purchased from Megazyme Ltd. (Bray, Ireland). The water soluble fraction of arabinoglucuronoxylan (oat spelt) was produced as described previously [85]. Acetylated galactoglucomannan (AcGGM, spruce) was a kind gift from Prof. Kirsi Mikkonen. Benzyl-d-glucuronate (BnGlcA) was purchased from Carbosynth® (Newbury, UK). The two acetylated xylobioses X2Ac5 (2,3-di-*O*-acetyl-β-d-Xyl*p*-(1,4)-1,2,3-tri-*O*-acetyl-α-d-Xyl*p* and X2Ac4 (2,3-di-*O*-acetyl-β-d-Xyl*p*-(1,4)-2,3-di-*O*-acetyl-d-Xyl*p*) were synthesized in house at Toulouse Biotechnology Institute (Additional file 4). The acetylated and feruloylated xylooligosaccharides (AcFaXOS) from corn fiber were a kind gift from Prof. Mirjam Kabel (University of Wageningen, Fraction B of [11]). All other used chemicals were ordered from Sigma-Aldrich (St. Louis, MO, USA).

### Heterologous protein expression and purification

The predicted signal sequence of BD-FAE (first 19 aa, Gram-negative bacteria) was removed and the sequence was codon optimized for expression in *E. coli* BL21 (DE3) (NEW ENGLAND BioLabs Inc., Ipswich, MA, USA) before gene synthesis into a pET29b(+) vector containing a C-terminal His-Tag for purification (GenScript USA Inc., Piscataway, NJ, USA). A plasmid containing a truncated form (∆Met1-Pro7) of BD-FAE was created and both plasmids were used for heat-shock transformation separately. Each strain was incubated in 500 mL MagicMedia™ (Thermo Fisher Scientific Inc., Waltham MA, USA) at 30 °C while shaking at 220 rpm for 20 h. Cells were harvested (20 min, 5000 rpm at 4 °C), suspended in lysis buffer (20 mM HEPES, pH 7.4) and frozen at -80 °C. After defrosting, cells were lysed by sonication on ice with a pulse of 2 s on/13 s off for 20 min at 37% amplitude (QSonica, Q500 Sonicator, microtip 1/16). The crude extract was clarified by centrifugation (20 min, 15,000 rpm at 4 °C) and filtration (0.45 µM Whatman™ filter) before purification with an ÄKTA system (GE Healthcare, Chicago, IL, USA). A 6 mL Ni–NTA column was equilibrated (20 mM HEPES, pH 7.4, 500 mM NaCl) with a flow rate of 1.0 mL/min. The flow rate was maintained for all following steps. Protein was loaded using a sample pump. The column was washed until the signal for protein detection by UV stabilized. Bound protein was eluted with a linear gradient of ten column volumes (0–100%, 20 mM HEPES, pH 7.4, 500 mM NaCl, 500 mM imidazole). The fractions containing the desired proteins were collected and desalted by buffer exchange (20 mM HEPES, pH7.4) using an Amicon® Ultra filter (10,000 MWCO, 15 mL). A second purification step was performed with a 1 mL HiTrap Q HP anion exchange column (GE Healthcare, Chicago, IL, USA) and a constant flow rate of 1.0 mL/min. After equilibrating the column (20 mM HEPES, pH 7.4) the sample was loaded, followed by a column wash (20 mM HEPES, pH 7.4). Protein was eluted with a linear gradient of 10 column volumes (0–100%, 20 mM HEPES, pH 7.4, 1 M NaCl). The purified proteins were desalted and concentrated for storage in the same manner as mentioned above. Protein concentrations were measured with Pierce BCA Protein Assay (Thermo Fisher Scientific Inc., Waltham MA, USA) and purity of the proteins were determined by SDS-PAGE and ultra-high-resolution Fourier transform-ion cyclotron resonance mass spectrometry (FT-ICR-MS, for method see next paragraph) (Additional file 2: Figure S1A, S2A).

### Determination of the quaternary structure

The oligomerization states of BD-FAE and its truncated form were determined. Native mass spectrometry was carried out with Bruker SolariX 12 T ultra-high-resolution FT-ICR-MS combined with Electrospray Ionization source. The storage buffer of the sample was exchanged to 10 mM ammonium acetate with a PD 10 column (GE Healthcare, Chicago, IL, USA) before injecting 70 µM sample into FT-ICR-MS at a flow rate of 250 µL/min. The inlet temperature was 353 K. Size exclusion chromatography on a 120 mL HiLoad 16/600 Superdex 200 column (GE Healthcare, Chicago, IL, USA) was performed with the ÄKTA system. BSA (Mw 66.5 kDa) was used as a standard. The hydrodynamic radius of the protein particles was studied with dynamic light scattering. Measurements were performed with DynaPro99 dynamic light scattering system (Wyatt Technology Corp.) with temperature-controlled micro sampler. The sample was filtered and measured by 20 scans.

### Determination of pH optima and kinetic parameters K_m_ and v_max_

The pH optimum of BD-FAE was tested in a pH range of 4.0–8.0 (sodium citrate buffer: pH 4.0–5.5, sodium phosphate buffer: pH 6.0–7.0, HEPES pH 7.5–8.0), using 0.4 µg enzyme and 1 mM 1-naphthyl acetate as substrate (stable in the given pH range) in a final reaction volume of 200 µL. The reaction was mixed in a 96-well plate and incubated at 40 °C while shaking at 350 rpm for 30 min. The hydrolysis into acetic acid and 1-naphthol was detected as increasing absorbance at 321 nm.

The kinetic parameters K_m_ and V_max_ for BD-FAE were determined on *p*NP-Ac (commonly used for kinetics) as substrate. A 500 mM *p*NP-Ac stock solution was dissolved in 100% DMSO (final DMSO content was 2.5%). The reactions were conducted in 50 mM sodium phosphate buffer at pH 6.0 with an enzyme dose varying between 1–4 µg and a *p*NP-Ac concentration varying between 1–10 mM in a final reaction volume of 200 µL. Incubation was conducted in a 96-well plate. Initially, the substrate was fully dissolved by shaking for 10 min at 40 °C followed by enzyme addition to start the reaction. The release of *p*NP from *p*NP-Ac was measured spectrophotometrically at 405 nm. The initial reaction rates (v_0_) were plotted against the corresponding initial *p*NP-A*c* concentrations to obtain a Michaelis–Menten curve, which was fitted by using a substrate inhibition equation in Origin 9.0 software.

### Initial screening

The initial screenings were performed in 96-well plates in a total volume of 200 µL. Samples were tested in triplicates, standards, substrate blanks and enzyme blanks in duplicates. For all *p*NP-glycosides and *p*NP-esters, 50 mM stock solutions in DMSO were prepared which were diluted to 1.25 mM in three different 50 mM buffers (sodium acetate buffer—pH 5.5, HEPES—pH 7.0, HEPES—pH 8.5, 160 µL). After adding 100 µg BD-FAE (40 µL) the final substrate concentration was 1 mM except of pNP-A. There the final concentration was 0.3 mM. For each polymeric substrate, 10 mg/mL stock solutions in water were prepared, which were diluted to 0.75 mg/mL in three different 50 mM buffers (160 µL) and mixed with 10% (g enzyme / g dry matter substrate) BD-FAE (40 µL) leading to a final substrate concentration of 0.6 mg/mL mixed with 12 µg enzyme. All plates were covered with an aluminum sealing and incubated at 40 °C, 300 rpm shaking for 2 h, 4 h and 24 h. To all incubations on *p*NP-glycosides and *p*NP-esters 50 µL of 500 mM Na_2_CO_3_ was added and absorbance was measured at 405 nm. Reactions on polymeric substrates was stopped by boiling for 10 min and catalytic activity was measured by PAHBAH-based reducing ends assay as previously described [86, 87]. After subtracting the absorbance of enzyme blank and buffer blank, the absorbance of the substrate blank was compared to the absorbance of the incubation of enzyme and substrate.

### Biochemical characterization

AcXE activity of BD-FAE was tested on (*p*NP-Ac in initial screening), X2Ac5, X2Ac4, AcGX, AcGGM, and AcFaXOS. FAE activity was tested on *p*NP-Fa and on AcFaXOS. GE activity was tested on BnGlcA. These experiments were performed in 5–50 mM HEPES (pH 7.0) at 40 °C and an enzyme dose of 1–3% (g enzyme / g dry matter substrate). Exceptions were reactions with AcGX and AcGGM, where reactions with AcGX were performed in 10 mM sodium citrate buffer (pH 6.0) at 40 °C and an enzyme dose of 1.5–4.5% (g enzyme / g dry matter substrate) and reactions with AcGGM were performed in 50 mM phosphate buffer (pH 6.0) at 30 °C and an enzyme dose of 0.75% (g enzyme / g dry matter substrate)*.* Incubation times varied between 0.5 h-24 h and are indicated in the corresponding graphs. The final substrate concentrations were as followed: X2Ac5 and X2Ac4: 1 mg/mL, AcGX: 15 mg/mL, AcGGM: 10 mg/mL, *p*NP-Fa: 1 mM, AcFaXOS: 10 mg/mL, and BnGlcA: 10 mM. The release of acetic acid from AcGX and AcGGM and the release of glucuronic acid from BnGlcA were analyzed with the acetic acid kit (K-ACETRM) and the glucuronic acid kit (K-URONIC), respectively. The release of *p*NP from *p*NP-Fa was measured spectrophotometrically at 405 nm and the release of acetic acid and ferulic acid from X2Ac5, X2Ac4 and AcFaXOS was analyzed using MALDI-TOF–MS. Total acetic acid release of AcGX was determined by complete alkaline saponification [88]. In this case, 100 µg of substrate was incubated in 200 µL of 0.1 M NaOH for 24 h at 30 °C while shaking at 400 rpm. The solution was neutralized with 1 M HCl before acetic acid determination via K-ACETRM kit. Absorbance values of enzyme incubations, enzyme and buffer blanks for the samples shown in Fig. 5A-B are given in Additional file 2: Table S4.

### MALDI-TOF-MS

An MDS Sciex matrix-assisted laser desorption/ionization time-of-flight mass spectrometry (MALDI-TOF MS) system was used equipped with a nitrogen laser of 337 nm. The measurements were conducted in positive and reflectron mode. The laser intensity was set to 80%, the mass range to 300–1800 Da, the focus mass to 1500 Da. The system was calibrated with xylooligosaccharides (300–1800 Da). For the sample preparation all chemicals had MS-grade. All samples were desalted (AG 50 W-X8 resin; Bio-Rad Laboratories, Hercules, CA, USA) and filtered (0.2 µM, Sartorius, Göttingen, GE). Subsequently, 1 µL sample was mixed with 1 µL of saturated 2,5-dihydroxybenzoic acid solution (10 mg/mL in 3:7 acetonitrile:water, Bruker Daltonics, Bremen, GE) on a metal target plate. The drops were dried under continuous air flow. Each sample was measured in duplicates and each spectrum is accumulated from at least six different spots. The obtained spectra were processed with mMass (www.mmass.org).

### ^1^H-NMR

The substrate blanks and enzyme incubations of BD-FAE (6 µg) on X2Ac5 (200 µg) in 10 mM HEPES buffer pH 7.0 (40 °C, 24 h, n = 2) used for MALDI-TOF analysis were freeze dried. Afterwards the recovered solid was suspended in 500 µL of CDCl_3_ and ^1^H-NMR spectra were obtained using a Bruker Avance Neo system at 600 MHz with a bbfo smartprobe (298 K, 20 s delay, 16 scans). The obtained spectra were processed in Mestrenova.

### Crystallization, data collection and structure solution

BD-FAE was crystallized at 20 °C by the hanging drop vapor diffusion method using 24-well plates (Greiner CELLSTAR) and siliconized cover slides (Hampton research). Crystals were obtained using crystallization solution consisting of 0.2 M ammonium sulfate, 25–30% polyethylene glycol monomethyl ether (PEG MME) 5000 and 0.1 M MES at pH 6.0. A 4 μL drop, including 2 μL of protein (12 mg/mL) and 2 μL of crystallization solution, was allowed to equilibrate against 500 μl of crystallization solution per well. Both full-length and truncated forms were crystallized under the same conditions. Thin needle like crystals were obtained within a week. They were cryoprotected with 30% ethylene glycol. Crystals were mounted in nylon loops and plunged into liquid nitrogen prior to data collection. Data collection for BD-FAE (PDB 6TKX) was carried out at Diamond Light Source on beamline i04 and for truncated form (PDB: 6XYC) at ESRF on beamline ID23-1. The data sets were processed and scaled with *XDS* [89]. The structure was solved using the automated molecular replacement and model building software phenix.mr_rosetta [90]. As templates the 250 closest structural homologs of the Protein Data Bank (PDB) obtained with a HHPred multiple sequence alignment (MPI Bioinformatics Toolkit [91]) were used. A clear molecular replacement solution was found with lowest Rfree of 0.357 which was refined with phenix.refine [90] and manual editing in Coot [92]. The structure of truncated BD-FAE was solved using the crystal structure of BD-FAE as template. In an attempt to obtain a complex structure with a bound XOS ligand, the BD-FAE crystals were soaked with 30 mM xylobiose, 30 mM xylotriose or 10 mM xylopentose. Binding of XOS was also studied by docking d-xylose, xylobiose, xylotetraose and xylohexaose into BD-FAE (PDB: 6TKX) with AutoDock Vina (ADV1.1.2, http://www.vina.scripps.edu/ [93]). The receptor molecules used in were the monomeric form of BD-FAE and two BD-FAE molecules packed in a way they packed in the crystal. For docking FA-Ara*f* into 6TKX, the ligand was built in 3D in CS ChemBioDraw Ultra and energy-minimized in UCSF Chimera [94].

## Supplementary Information

**Additional file 1: Table S1.** Spreadsheet of genetic polysaccharide utilization loci (PUL) context of BD-FAE and its 200 closest homologs obtained by BLASTp search against internal PULDB. Locus tags were completed with BLASTp bits score, e-values, sequence similarities and CAZyme specificities in the genomic context, based on the predicted PUL and human curation.

**Additional file 2. **Additional data of BD-FAE biochemical characterization including **Table S2.** List of all *p*NP-glycosides, *p*NP-Ac and polymeric substrates used for initial screening of BD-FAE. **Figure S1. (A)** SDS-PAGE of purified BD-FAE and its truncated form, **(B)** pH optimum of BD-FAE, and **(C)** kinetic parameters of BD-FAE. **Figure S2.** Oligomerization state of BD-FAE **(A)** by native mass spectrometry, **(B)** by dynamic light scattering, and **(C + D)** by size exclusion chromatography. **Figure S3.** Initial screening of BD-FAE on *p*NP-glycosides and *p*NP-Ac. **Table S3.** Comparison of kinetic parameters of carbohydrate esterases on *p*NP-acetate. **Figure S4.** MALDI-TOF spectra before **(A)** and after **(B)** incubating BD-FAE on X2Ac4. **Figure S5.** Glucuronoyl esterase activity of BD-FAE. **Table S4.** Average absorbance values for enzyme incubation, buffer-, enzyme -, and substrate blanks corresponding to the results of photometric assays shown in Fig. 5A-B.

**Additional file 3. **Additional data on BD-FAE’s crystal structure including **Table S5.** Data processing and refinement statistics of BD-FAE (PDB: 6TKX) and its truncated form (PDB: 6XYC). **Figure S6.** Active site of BD-FAE with **(A)** sulfate ion, **(B)** protease inhibitor AEBSF as ligands and **(C)** an aligned version. **Figure S7.** Comparison of BD-FAE overall structure to other carbohydrate esterases.

**Additional file 4. **Additional data on the synthesis of per-acetylated xylobioses, X2Ac5 and X2Ac4 including protocol, characterizations, and **Figures S8–S11.** ^1^H and ^13^C NMR spectra.

## Data Availability

All data generated or analyzed during this study are included in this published article and its Additional files. The sequences and the crystal structures of BD-FAE (PDB: 6TKX) and its truncated form (PDB: 6XYC) are deposited in PDB.
