# Supplementary material for: Polysaccharide utilization loci-driven enzyme discovery reveals BD-FAE: a bifunctional feruloyl and acetyl xylan esterase active on complex natural xylans
Source: Biotechnol Biofuels. 2021 May 31;14:127. doi: 10.1186/s13068-021-01976-0 (PMC8165983; doi:10.1186/s13068-021-01976-0)
Supplement: Supplementary file 2 — Additional file 2. Additional data of BD-FAE biochemical characterization including Table S2. List of all pNP-glycosides, pNP-Ac and polymeric substrates used for initial screening of BD-FAE. Figure S1. (A) SDS-PAGE of purified BD-FAE and its truncated form, (B) pH optimum of BD-FAE, and (C) kinetic parameters of BD-FAE. Figure S2. Oligomerization state of BD-FAE (A) by native mass spectrometry, (B) by dynamic light scattering, and (C + D) by size exclusion chromatography. Figure S3. Initial screening of BD-FAE on pNP-glycosides and pNP-Ac. Table S3. Comparison of kinetic parameters of carbohydrate esterases on pNP-acetate. Figure S4. MALDI-TOF spectra before (A) and after (B) incubating BD-FAE on X2Ac4. Figure S5. Glucuronoyl esterase activity of BD-FAE. Table S4. Average absorbance values for enzyme incubation, buffer-, enzyme -, and substrate blanks corresponding to the results of photometric assays shown in Fig. 5A-B. [file 13068_2021_1976_MOESM2_ESM.docx]

## Additional File 2 – Additional data on biochemical characterization of BD-FAE

**
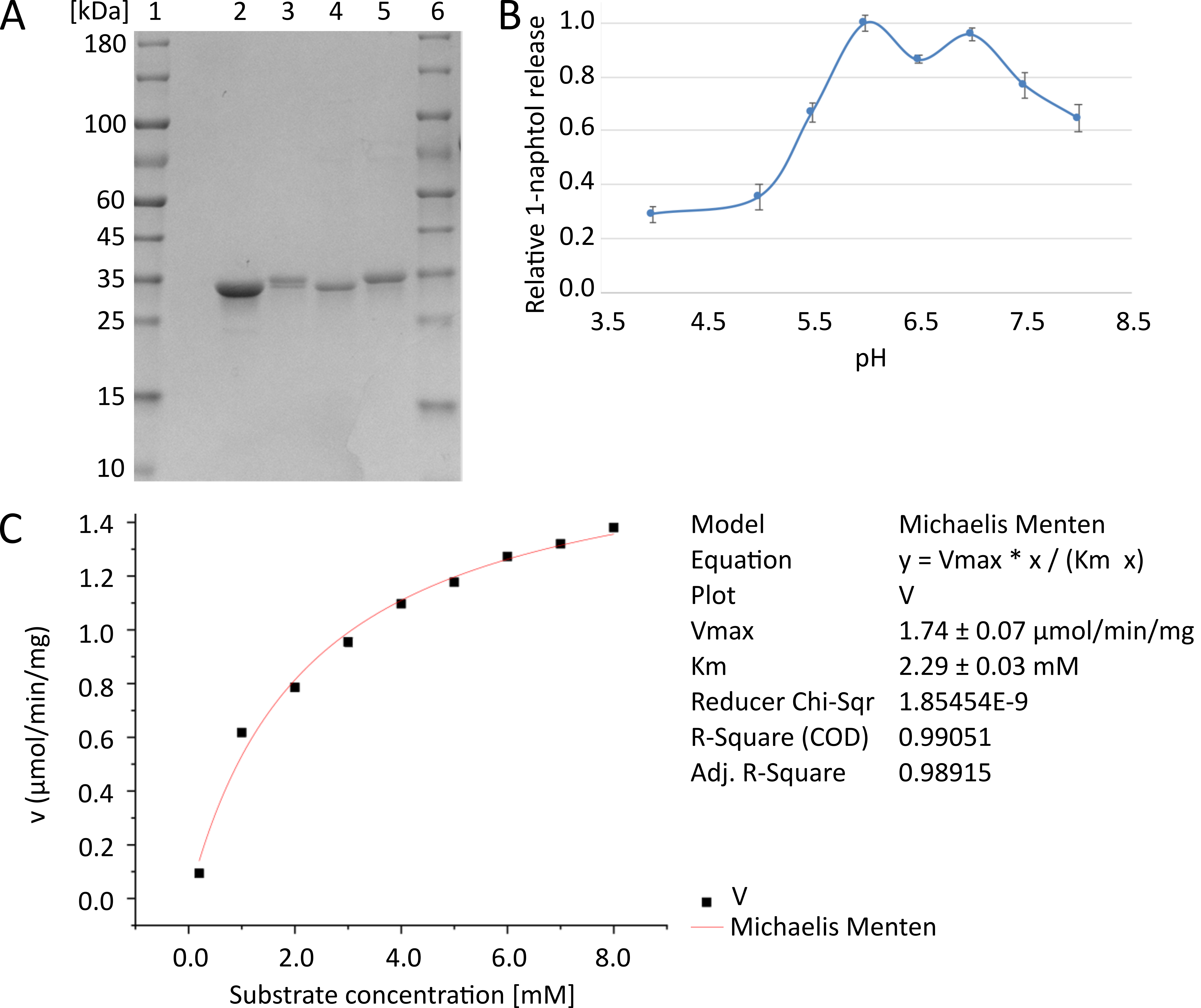
**

**Figure S1 (A)** SDS-PAGE of purified BD-FAE. (1, 6) molecular weight standard, (2) truncated BD-FAE after Ni-NTA purification, (3) BD-FAE after Ni-NTA purification, (4) truncated BD-FAE after anionic exchange chromatography purification, (5) BD-FAE after anionic exchange chromatography purification. **(B)** The pH optimum of BD-FAE. Relative activity of 1 % (w/w, 0.4 µg) BD-FAE on 1 mM 1-naphtyl acetate at a pH range of 4.0 – 8.0 at 40°C after 0.5h to determine pH optimum; n=8. **(C)** Kinetic parameters obtained after incubating 1-4 µg BD-FAE on 1-10 mM *p*NP-acetate at pH 6.0 and 40°C for 10 min. Michaelis-Menten curve was obtained by plotting initial reaction rates against corresponding initial *p*NP-acetate concentrations by using the substrate inhibition equation fit in Origin 9.0. 1-naphtol and *p*NP release during pH optimum and kinetic parameter assays were monitored by measuring absorbance at 321 nm and 405 nm, respectively.

**
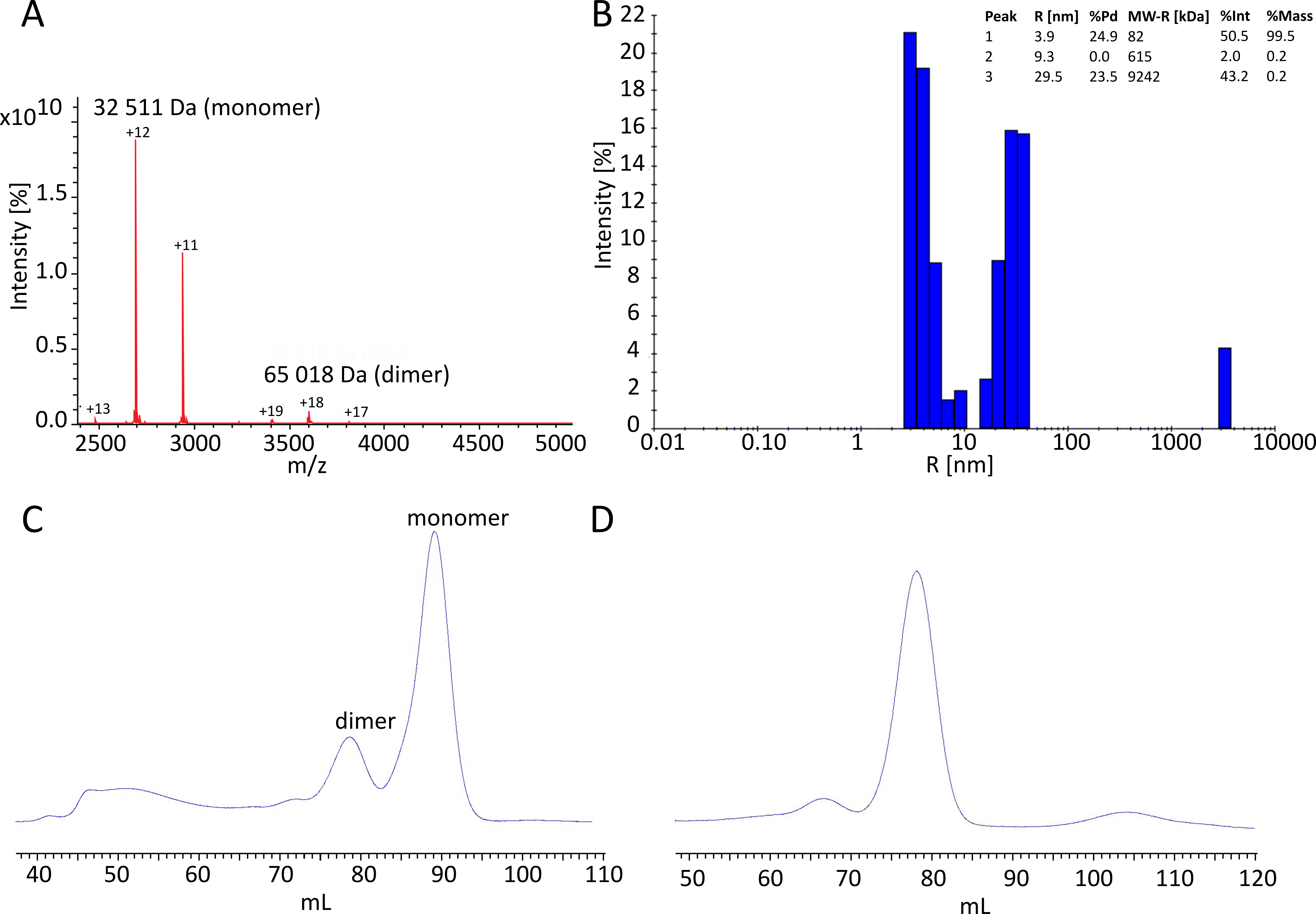
Figure S2** **(A)** Native mass spectrometry spectrum. The charge states are marked on top of each peak. Each oligomerization state gives three charge states and the native mass calculated for the oligomers are shown on top of them. 32 511 Da for a monomer and 65 018 Da for a dimer. **(B)** Dynamic light scattering result. **(C)** Size exclusion chromatography run of BD-FAE. **(D)** Size exclusion chromatography run for BSA (Mw= 66 463 Da) as a standard.

**Table S2** List of all substrates used for the initial screening of BD-FAE.

| ***para-*nitrophenyl-substituted substrates** | **Polymeric samples** |
| --- | --- |
| *p*NP-α-d-galactopyranoside | Wheatarabinoxylan (medium viscosity) |
| *p*NP-β-d-galactopyranoside | Arabinoglucuronoxylan (Oat spelt, water soluble fraction ^1^) |
| *p*NP-α-d-glucopyranoside | Glucuronoxylan (Beech wood) |
| *p*NP-β-d-glucopyranoside | Carboxymethyl-cellulose (4M) |
| *p*NP-α-l-arabinofuranoside | Dextran |
| *p*NP-β-d-mannopyranoside | Pullulan |
| *p*NP-β-d-xylopyranosde | Lichenan (Icelandic Moss) |
| *p*NP-α-l-fucopyranoside | Galactoxyloglucan (Tamarind, high viscosity) |
| *p*NP-β-d-cellobioside | Mannan |
| *p*NP-acetate | Arabinogalactan (Larch wood) |
|  | Glucomannan (Konjac, low viscosity) |
|  | Galactomannan (Guar, medium viscosity) |
|  | Galactan (Lupin) |
|  | Pectic-galactan (Lupin) |
|  | Rhamnogalacturonan (Soy Bean) |
|  | Rhamnogalacturonan I (Potato) |
|  | Arabinan (Sugar beet) |

**

**

**Figure S3** Initial screening of BD-FAE. 100 µg enzyme incubated on 1mM *p*NP-substituted substrates (200 µL final volume) at 40°C and three different pHs (5.5, 7.0 and 8.5). Absorbance measured at 405 nm at three time points (2h, 4h, 24h), n = 3.

**Table S3** Comparison of kinetic parameters of carbohydrate esterases on *p*NP-acetate.

| **Species** | **Enzyme name** | **Accession number** | **Substrate** | **k_cat_ [s^-1^]** | **Reference** |
| --- | --- | --- | --- | --- | --- |
| Beaver dropping metagenome ^2^ | Bifunctional AcXE/ FAE, BD-FAE | PDB number: 6TKX | *p*NP-acetate | 0.89 | this study |
| *Butyrivibrio proteoclasticus* | Promiscuous FAE, Est1E | PDB number: 2WTM_A | *p*NP-acetate | 23 | Goldstone et al., 2010 |
| *Geobacillus stearothermophilus* | AcXE Axe2 | UniProtKB/TrEMBL accession number Q09LX1 | *p*NP-acetate | 31 | Alalouf et al., 2011 |
| *Flavobacterium johnsoniae* | AcXE, FjoAcXE | Genbank ID: ABQ06890.1 | *p*NP-acetate | 121 | Razeq *et al.*, 2018 |
| *Butyrivibrio proteoclasticus* | AcXE, Est2A | Genbank ID: CP001810 | *p*NP-acetate | 139 | Till et al., 2013 |


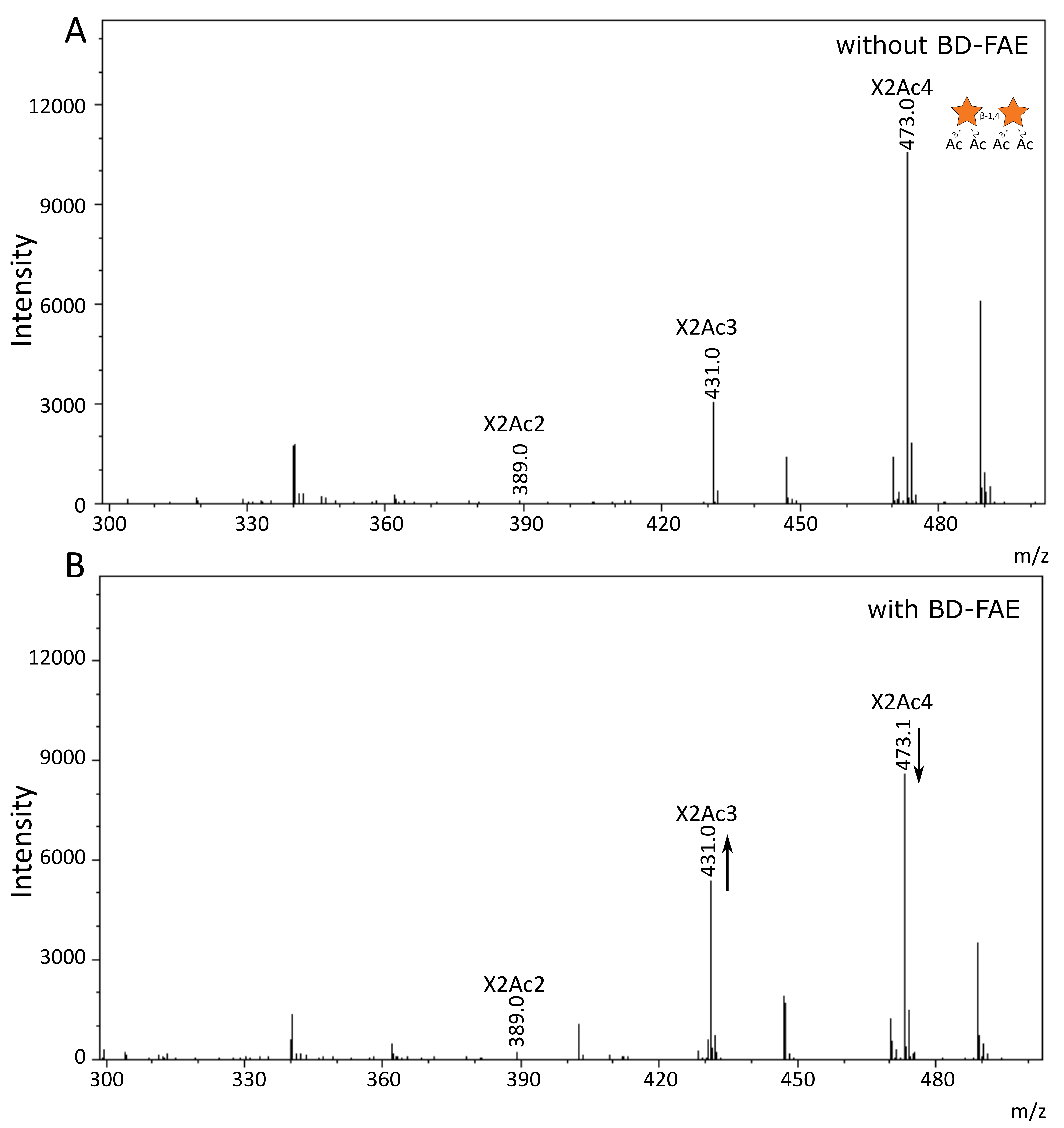
**Figure S4** MALDI-TOF spectra **(A)** before and **(B)** after incubating 3% (g enzyme / g dry matter substrate) BD-FAE on X2Ac4 (2,3-di-*O*-acetyl-β-d-Xyl*p*-(1,4)-2,3-di-*O*-acetyl-α-d-Xyl*p*) (1 mg/mL final concentration) at pH 7.0 and 40°C for 24h. All m/z values are sodium adducts. X = orange star = xylosyl, Ac = acetyl residues (n = 2).


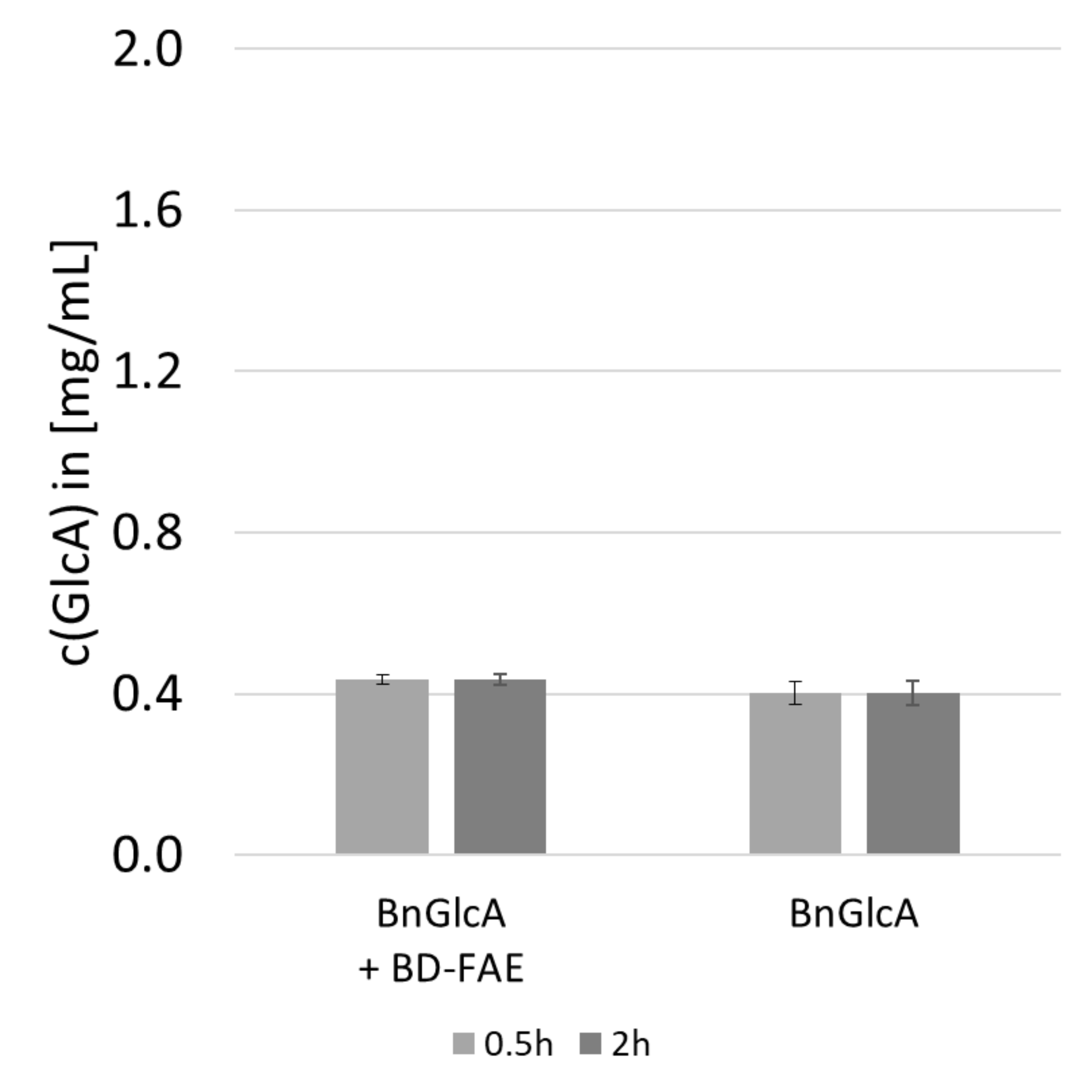
**Figure S5** Glucuronoyl esterase activity of BD-FAE. 1,4 µg enzyme incubated on 10 mM benzyl-d-glucuronate (BnGlcA, 500 µL final volume) at 40 °C and pH7 (5 mM HEPES ), analyzed with glucuronic acid kit (K-URONATE), n = 2.

**Table S4** Average of absorbance values for the enzyme incubation, buffer, enzyme, and substrate blanks corresponding to the photometric assay results shown in Figure 5A-B. For samples measured with K-ACETRM the ∆Abs (A1 – A2) is given.

| **Figure** | **Assay** | **Sample** | **Wavelength [nm]** | **Absorbance** |
| --- | --- | --- | --- | --- |
| 5A | *p*NP | 50 mM HEPES pH 7.0 incubated for 2h (n=2) | 405 | 0.0391 ± 0.001 |
| 5A | *p*NP | 1 µg BD-FAE in 50 mM HEPES pH 7.0 incubated for 2h (n=3) | 405 | 0.0410 ± 0.001 |
| 5A | *p*NP | 1 mM *p*NP-ferulate in 50 mM HEPES pH 7.0 incubated for 2h (n=6) | 405 | 0.2596 ± 0.018 |
| 5A | *p*NP | 1mM *p*NP-ferulate + 1 µg BD-FAE in 50 mM HEPES pH 7.0 incubated for 2h (n=6) | 405 | 1.0104 ± 0.024 |
| 5B | K-ACETRM | 10 mM sodium citrate buffer pH 6.0 incubated for 2h (n=2) | 340 | 0.0527 ± 0.053 |
| 5B | K-ACETRM | 4.5 % w/w BD-FAE (335 µg) in 10 mM sodium citrate buffer pH 6.0 incubated for 2h (n=3) | 340 | 0.1901 ± 0.023 |
| 5B | K-ACETRM | 7.5 mg AcGX in 10 mM sodium citrate buffer pH 6.0 incubated for 2h (n=3) | 340 | 0.0758 ± 0.026 |
| 5B | K-ACETRM | 7.5 mg AcGX + 335 µg BD-FAE in 10 mM sodium citrate buffer pH 6.0 incubated for 2h (n=6) | 340 | 0.5645 ± 0.221 |

### References

**1**. Martens, E. C. *et al.* Recognition and degradation of plant cell wall polysaccharides by two human gut symbionts. *PLoS Biol.* **9**, e1001221 (2011).

**2**. Wong, M. T. *et al.* Comparative metagenomics of cellulose- and poplar hydrolysate-degrading microcosms from gut microflora of the Canadian beaver (*Castor canadensis*) and North American moose (*Alces americanus*) after long-term enrichment. *Front. Microbiol.* **8:2504**, (2017).

**3**. Goldstone, D. C. *et al.* Structural and functional characterization of a promiscuous feruloyl esterase (Est 1E) from the rumen bacterium Butyrivibrio proteoclasticus. *Proteins Struct. Funct. Bioinforma.* **78**, 1457–1469 (2010).

**4**. Alalouf, O. *et al.* A new family of carbohydrate esterases is represented by a GDSL hydrolase/acetylxylan esterase from *Geobacillus stearothermophilus* *. *J. Biol. Chem.* **286**, 41993–42001 (2011).

**5**. Razeq, F. M. *et al.* A novel acetyl xylan esterase enabling complete deacetylation of substituted xylans. *Biotechnol. Biofuels* **11:74**, (2018).

**6**. Till, M. *et al.* Structure and function of an acetyl xylan esterase (Est2A) from the rumen bacterium Butyrivibrio proteoclasticus. *Proteins Struct. Funct. Bioinforma.* **81**, 911–917 (2013).
