# Supplementary material for: Polysaccharide utilization loci-driven enzyme discovery reveals BD-FAE: a bifunctional feruloyl and acetyl xylan esterase active on complex natural xylans
Source: Biotechnol Biofuels. 2021 May 31;14:127. doi: 10.1186/s13068-021-01976-0 (PMC8165983; doi:10.1186/s13068-021-01976-0)
Supplement: Supplementary file 3 — Additional file 3. Additional data on BD-FAE’s crystal structure including Table S5. Data processing and refinement statistics of BD-FAE (PDB: 6TKX) and its truncated form (PDB: 6XYC). Figure S6. Active site of BD-FAE with (A) sulfate ion, (B) protease inhibitor AEBSF as ligands and (C) an aligned version. Figure S7. Comparison of BD-FAE overall structure to other carbohydrate esterases. [file 13068_2021_1976_MOESM3_ESM.docx]

## Additional File 3: Additional information on BD-FAE’s crystal structure

**Table S5** Data processing and refinement statistics of solved crystal structures of BD-FAE (PDB: 6TKX) and its truncated form (PDB: 6XYC). Values for highest resolution shell are in parenthesis.

| Data processing | Full length | Truncated |
| --- | --- | --- |
| Beamline | i04, DLS | ID23-1, ESRF |
| Wavelength | 0.9795 | 0.91587 |
| Space group | P4_3_ 2_1_ 2 | P4_3_ 2_1_ 2 |
| Unit cell (a,b,c, ) | 108.5 108.5 44.8 | 107.6 107.6 44.4 |
| Unique reflections | 17058 (1691) | 22923 (2232) |
| Rmeas (%) | 14.3 (150) | 14.6 (169.7) |
| I/sigma | 10.0 (1.0) | 11.3 (1.2) |
| CC1/2 | 99.8 (45.5) | 99.8 (51.5) |
| Completeness | 99.8 (99.9) | 99.9 (99.4) |
| Multiplicity | 8.7 (9.0) | 7.0 (6.9) |
| Structure refinement: |  |  |
| Resolution (Å) | 2.06 | 1.85 |
| Rwork/Rfree (%) | 20.5/27.0 | 17.6/20.3 |
| No. of atoms: |  |  |
| Non-hydrogen atoms | 2337 | 2367 |
| Protein | 2217 | 2166 |
| Ligand | 5 | 12 |
| Solvent | 115 | 189 |
| Mean B factors (Å^2^): |  |  |
| Protein | 44.0 | 29.3 |
| Ligand | 51.0 | 35.6 |
| Solvent | 42.5 | 34.3 |
| r.m.s.d. |  |  |
| Bond lenght (Å) | 0.012 | 0.004 |
| Bond angles (˚) | 1.43 | 0.70 |
| Ramachandran favored (%) | 95.17 | 95.76 |
| Ramachandran outliers (%) | 0.34 | 0.71 |
| PDB code | 6TKX | 6XYC |


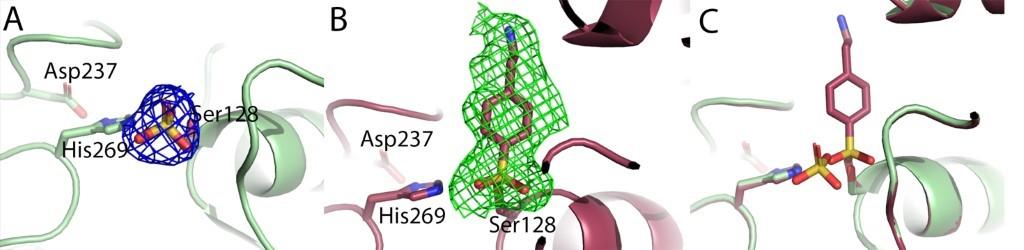


**Figure S6** The active site of BD-FAE. **(A)** A sulfate ion was refined in the active site of BD-FAE. The 2Fo-Fc density is shown at 1.0σ contour level. **(B)** In truncated BD-FAE, the protease inhibitor AEBSF was found to be covalently bound to Ser128. A polder map calculated by emitting AEBSF is shown is green at 3.0σ contour level. **(C)** Aligned structures of BD-FAE and its truncated form show that the ligands are positioned slightly differently and Ser128 is bound to AEBSF but not to the sulphate ion.

## Comparison of BD-FAE’s primary & tertiary structure with other carbohydrate esterases

Many members of the CE family share an α/β-hydrolase fold, however, proteins with similar primary or tertiary structures do not necessarily have similar catalytic activities and vice versa ^1–3^. To investigate whether any conserved regions can be identified between the crystal structures of BD-FAE and other CEs, four structurally close enzymes were superimposed (Figure S7). Namely, the three closest hits of a BLASTp search against PDB (accessed 08.2020), being a bifunctional GH43-CE of *Bacteroides eggerthii* (PDB: 6MLY), a characterized bifunctional acetyl esterase/xyloside hydrolase of *Caldicellulosiruptor lactoaceticus* (*Cl*H10, PDB: 6AOA ^4^) and characterized thermophilic esterase from *Thermogutta terrifontis* (*Tt*Est2, PDB: 5AO9 ^5^). Their query covers were 95%, 81% and 81% and their sequence identities were 55%, 27%, and 26%, respectively*.* The fourth superimposed crystal structure belonged to the characterized fungal FAE of *Anaeromyces mucronatus* (*Am*CE1/Fae1A, PDB: 5CXX ^6,7^).

The overall crystal structures of BD-FAE and the CE domain of 6MLY were found to be quite similar, yet sharing only 55% sequence similarity. In particular, the small β-hairpin found in BD-FAE was also observed in 6MLY with the same topological position (Figure S7A). Moreover, the disulfide bond forming cysteines at the active site of BD-FAE (Cys186- Cys244) were also found in 6MLY. However, in the electron density map of the 6MLY crystal, they were found as free cysteines. Unfortunately, there is no biochemical characterization of 6MLY available that could be compared to BD-FAE. *Cl*H10, *Tt*Est2, and *Am*CE1, however, are biochemically characterized but their crystal structures show very little similarities as their low sequence identities already suggest (Figure S7B-D). Neither the N-terminal tail, nor the small β-hairpin described for BD-FAE are present. The loop following the β1-strand was ten residues shorter in BD-FAE if compared to ClH10. In the crystal structures of *Tt*Est2 and BD-FAE two small α-helices (α4 and α5) after β6-strand were found that could be superimposed well (Figure S7C). Otherwise, both crystal structures differed drastically e.g. by the N-terminus from Gln2 to Asn33, the region of small β-sheet from Thr140 to Leu163 and the loop region from Met181 to Asp211 (Figure S7C). *Tt*Est2 belongs to the CE3 family in which the enzymes have a characteristic “cap” domain covering the active site. *Tt*Est2, however, only has a minimal cap domain. In BD-FAE there is no cap domain although the N-terminal tail could possibly turn close to the active site in the monomeric form of BD-FAE for energy minimization. This is not observed in the crystal structure, but the high temperature factors of the N-terminal residues indicate the flexibility of the terminus.

Additionally, to the sequence based BLASTp search against PDB, structurally similar proteins were searched through the DALI-server ^8^ (accessed on 21.10.19). The most similar protein was again 6MLY with an r.m.s.d. value of 1.7. The three following hits were tannin acyl hydrolase from *Lactobacillus plantarum* (PDB: 4J0D, r.m.s.d. value of 2.5), esterase/lipase from *Lactobacillus rhamnosis* (PDB: 4N5H, r.m.s.d. value of 2.6) and lipase from *Enterobacter asburiae* (PDB: 6KMO, r.m.s.d. value of 2.4). It is worthy to notice, that the predicted catalytic functions of the sequence-based hits were closer to the actual catalytic activity of BD-FAE than those of the structure-based hits. However, the huge variance of primary and tertiary structures within the CE family and the α/β-hydrolase fold once more indicated that neither can be used alone for functional prediction especially in times of exponentially growing numbers of sequenced genomes (GenBank® ^9^, accessed on 07.2020).


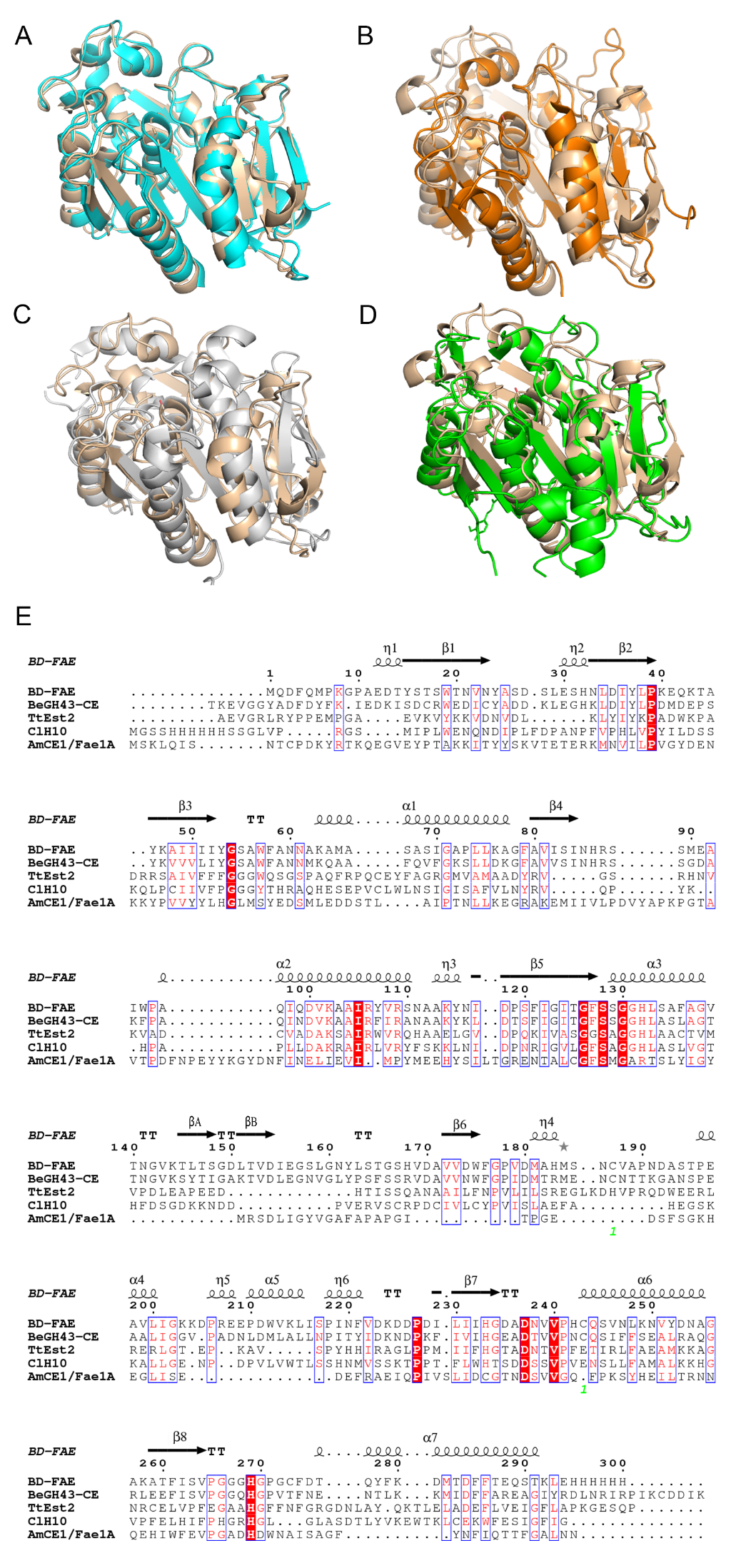


**Figure S7** BD-FAE overall structure (PDB: 6TKX, beige) was superimposed with **(A)** CE domain of bifunctional of GH43-CE of *Bacteroides eggerthii* (PDB: 6MLY, turquoise), **(B)** xyloside hydrolase and acetyl esterase from *Caldicellulosiruptor lactoaceticus* (*Cl*H10, PDB: 6A6O, orange), **(C)** thermophilic esterase from *Thermogutta terrifontis* (*Tt*Est2, PDB: 5AO9, gray), **(D)** ferulic acid esterase from *Anaeromyces mucronatus* (*Am*CE1, PDB: 5CXX, green). The catalytic serines are shown in sticks. **(E)** Multiple sequence alignment of all five sequences. Identical amino acid residues were highlighted in red and similar residues were boxed in blue and written in red. Green 1 indicates cysteines involved in disulfide bond formation.

**References**

1. Garron, M. L. & Henrissat, B. The continuing expansion of CAZymes and their families. *Curr. Opin. Chem. Biol.* **53**, 82–87 (2019).

2. Holmquist, M. Alpha/beta-hydrolase fold: Enzymes, structures, functions and mechanisms. *Curr. Protein Pept. Sci.* **1**, 209–235 (2005).

3. Lenfant, N. *et al.* Proteins with an alpha/beta hydrolase fold: Relationships between subfamilies in an ever-growing superfamily. *Chem. Biol. Interact.* **203**, 266–268 (2013).

4. Cao, H. *et al.* Structural insights into the dual-substrate recognition and catalytic mechanisms of a bifunctional acetyl ester-hyloside hydrolase from *Caldicellulosiruptor lactoaceticus*. *ACS Catal.* **9**, 1739–1747 (2019).

5. Sayer, C. *et al.* The structure of a novel thermophilic esterase from the Planctomycetes species, *Thermogutta terrifontis* reveals an open active site due to a minimal ‘cap’ domain. *Front. Microbiol.* **6:1294**, (2015).

6. Qi, M. *et al.* Isolation and characterization of a ferulic acid esterase (Fae1A) from the rumen fungus *Anaeromyces mucronatus* . *J. Appl. Microbiol.* **110**, 1341–1350 (2011).

7. Gruninger, R. J. *et al.* Contributions of a unique β-clamp to substrate recognition illuminates the molecular basis of exolysis in ferulic acid esterases. *Biochem. J.* **473**, 839–849 (2016).

8. Holm, L. Benchmarking fold detection by DaliLite v.5. *Bioinformatics* **35**, 5326–5327 (2019).

9. Benson, D. A. *et al.* GenBank. *Nucleic acids research* **41**, D36-42 (2013).
