# Supplementary material for: Polysaccharide utilization loci-driven enzyme discovery reveals BD-FAE: a bifunctional feruloyl and acetyl xylan esterase active on complex natural xylans
Source: Biotechnol Biofuels. 2021 May 31;14:127. doi: 10.1186/s13068-021-01976-0 (PMC8165983; doi:10.1186/s13068-021-01976-0)
Supplement: Supplementary file 4 — Additional file 4. Additional data on the synthesis of per-acetylated xylobioses, X2Ac5 and X2Ac4 including protocol, characterizations, and Figures S8–S11. 1H and 13C NMR spectra. [file 13068_2021_1976_MOESM4_ESM.docx]

## Additional File 4: Additional information on the synthesis of acetylated xylobioses, X2Ac5 and X2Ac4

## General

The evolution of the synthesis of acetylated xylobioses was monitored by analytical thin-layer chromatography using silica gel 60 F254 precoated plates (Merck KGaA, Darmstadt Germany). Spots were visualized by soaking the plates in a 0.1% w/v orcinol solution, containing a mixture of sulfuric acid/ethanol/water (3:72.5:22.5 v/v/v), followed by charring. Product purifications was achieved using a Reveleris® flash chromatography automated system equipped with prepacked silica gel cartridges (BUCHI, Villebon-sur-Yvette, France). Yields refer to chromatographically pure compounds. NMR spectra were recorded at 298K on a Bruker Avance II 500 spectrometer at 500 and 125 MHz for ^1^H and ^13^C respectively. Coupling constants (J) are reported in Hz, and chemical shifts (δ) are given in ppm with residual solvents signal as internal reference ^1^. Multiplicities are reported as follows: s = singlet, d = doublet, dd = doublet of doublets, t = triplet, m = multiplet, and br = broad. Analysis and assignments were performed using 1D (^1^H, ^13^C and Jmod) and 2D (COSY and HSQC) experiments. Roman numerals in ascending order are given to the residues from the reducing end. High-resolution mass spectra (HRMS) analyses were performed by the CRMPO (Centre régional de mesures physiques de l'Ouest, University of Rennes, France) in positive ionization mode (ES+) on a ThermoFisher Q-Exactive.

## 2,3-di-*O*-acetyl-β-d-xylopyranosyl-(1,4)-1,2,3-tri-*O*-acetyl-α-d-xylopyranoside (X2Ac5) and 2,3-di-*O*-acetyl-β-d-xylopyranosyl-(1,4)-2,3-di-*O*-acetyl-d-xylopyranose (X2Ac4)

Novozym® 435 (immobilized lipase from *Candida antarctica* B, 10.0 g) was added to a solution of 2,3,4-tri-*O*-acetyl-β-d-xylopyranosyl-(1,4)-1,2,3-tri-*O*-acetyl-d-xylopyranoside (2.672 g, 5.00 mmol) ^2,3^ in a THF/*tert*-amyl alcohol mixture (50 mL, 1:1 v/v). After stirring for 3 days at 37°C, the reaction was terminated by enzyme removal using filtration. The filter cake was washed with dichloromethane and the filtrate was concentrated by evaporation of the solvents at reduced pressure. Flash chromatography (using gradient dichloromethane/acetone, 1:0 to 8:2 v/v) afforded X2Ac5 (0.940 g, 1.91 mmol, 38%) ^4^ and then X2Ac4 (0.899 g, 2.00 mmol, 40%) as white powders.

**2,3-di-*O*-acetyl-β-d-xylopyranosyl-(1,4)-1,2,3-tri-*O*-acetyl-α-d-xylopyranoside (X2Ac5).** ^1^H NMR (CDCl_3_) δ 6.21 (1H, d, J_1,2_ = 3.7, H-1^I^), 5.38 (1H, dd, J_2,3_ = 10.1 and J_3,4_ = 9.3, H-3^I^), 4.96 (1H, dd, J_1,2_ = 3.7 and J_2,3_ = 10.1, H-2^I^), 4.85 (1H, t, J_2,3_ = J_3,4_ = 7.7, H-3^II^), 4.81 (1H, d, J_1,2_ = 5.8 and J_2,3_ = 7.7, H-2^II^), 4.52 (1H, d, J_1,2_ = 5.8, H-1^II^), 4.04 (1H, dd, J_4,5a_ = 4.5 and J_5a,5b_ = 11.9, H-5a^II^), 3.88-3.83 (1H, m, H-4^I^), 3.81-3.76 (2H, m, H-5a^I^ and H-4^II^), 3.67 (1H, t, J_4,5b_ = J_5a,5b_ = 11.1, H-5b^I^), 3.40 (1H, dd, J_4,5b_ = 7.8 and J_5a,5b_ = 11.9, H-5b^II^), 2.17, 2.09, 2.07, 2.05 and 2.01 (5 x 3H, 5s, C*H_3_*CO); ^13^C NMR (CDCl_3_) δ 171.3, 169.9, 169.8, 169.2 and 169.1 (CH_3_*C*O), 100.1 (C-1^II^), 89.2 (C-1^I^), 75.2 (C-4^I^), 74.3 (C-3^II^), 70.2 (C-2^II^), 70.0 (C-3^I^), 69.5 (C-2^I^), 67.9 (C-4^II^), 64.3 (C-5^II^), 61.3 (C-5^I^), 20.9, 20.9, 20.8, 20.7 and 20.5 (*C*H_3_CO).

**2,3-di-*O*-acetyl-β-d-xylopyranosyl-(1,4)-2,3-di-*O*-acetyl-d-xylopyranose (X2Ac4).** ^1^H NMR (CDCl_3_) δ 5.42 (0.7H, dd, J = 8.7 and 9.8, H-3^Iα^), 5.33 (0.7H, d, J_1,2_ = 3.5, H-1^Iα^), 5.16 (0.3H, t, J_2,3_ = J_3,4_ = 9.3, H-3^Iβ^), 4.85 (1H, t, J_2,3_ = J_3,4_ = 7.3, H-3^II^), 4.80-4.77 (1.7H, m, H-2^Iα^ and H-2^II^), 4.74 (0.3H, dd, J_1,2_ = 7.8 and J_2,3_ = 9.3, H-2^Iβ^), 4.64 (0.3H, br d, J_1,2_ = 7.8, H-1^Iβ^), 4.54 (0.7H, d, J_1,2_ = 5.7, H-1^IIα^), 4.53 (0.3H, d, J_1,2_ = 5.7, H-1^IIβ^), 4.05 (1H, dd, J_4,5a_ = 4.4 and J_5a,5b_ = 11.9, H-5a^II^), 3.99 (0.3H, dd, J_4,5a_ = 5.5 and J_5a,5b_ = 11.9, H-5a^Iβ^), 3.88-3.80 (1.7H, m, H-4^Iβ^, H-5a^Iα^ and H-4^Iα^), 3.79-3.74 (1H, m, H-4^II^), 3.70 (0.7H, t, J = 4.4 and 9.9, H-5b^Iα^), 3.40 (1H, dd, J_4,5b_ = 7.6 and J_5a,5b_ = 11.9, H-5b^II^), 3.33 (0.3H, dd, J_4,5b_ = 10.3 and J_5a,5b_ = 11.9, H-5b^Iβ^), 2.08-2.05 (12H, m, C*H_3_*CO); ^13^C NMR (CDCl_3_) δ 171.2-169.1 (CH_3_*C*O), 99.7 and 99.6 (C-1^IIα and β^), 96.0 (C-1^Iβ^), 90.3 (C-1^Iα^), 75.3 and 75.1 (C-4^Iα and β^), 74.0 and 73.9 (C-3^IIα and β^), 73.4 (C-2^Iβ^), 72.2 (C-3^Iβ^), 71.3 and 70.1 (C-2^Iα^ and C-2^II^), 69.9 (C-3^Iα^), 67.8 and 67.8 (C-4^II^), 64.2 and 64.1 (C-5^II^), 63.4 (C-5^Iβ^), 59.2 (C-5^Iα^), 20.9-20.7 (*C*H_3_CO).


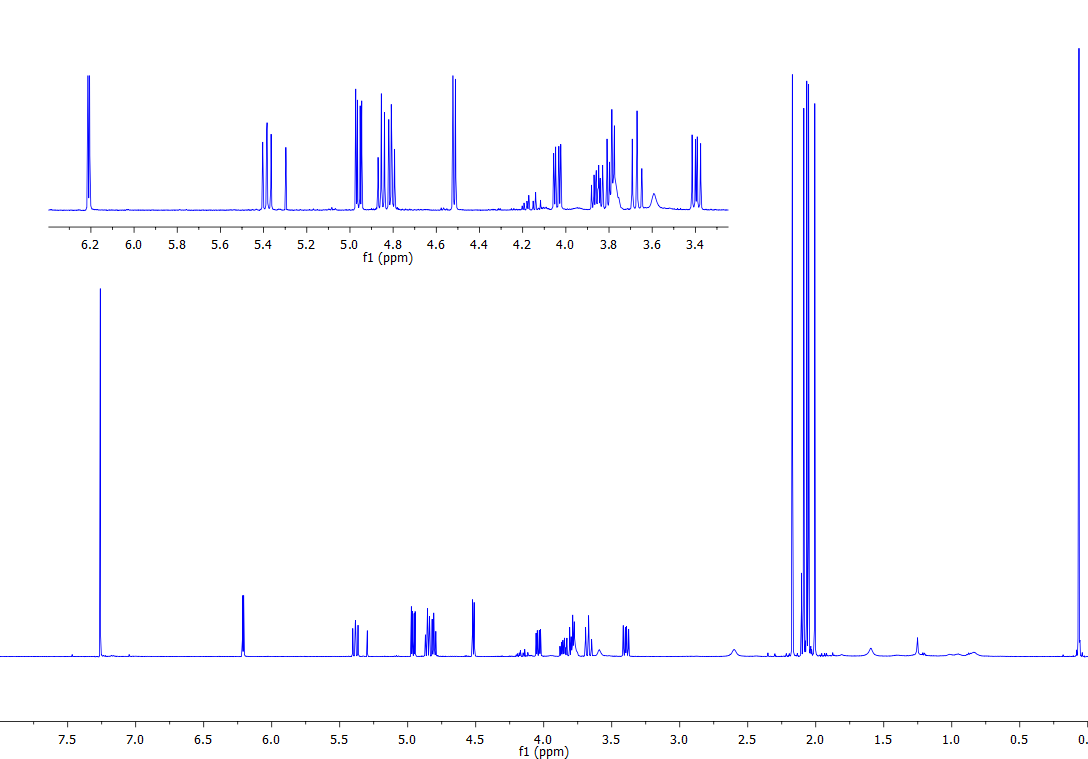


**Figure S8** ^1^H NMR spectrum (CDCl_3_, 298K) of 2,3-di-*O*-acetyl-β-d-xylopyranosyl-(1,4)-1,2,3-tri-*O*-acetyl-α-d-xylopyranoside (X2Ac5).
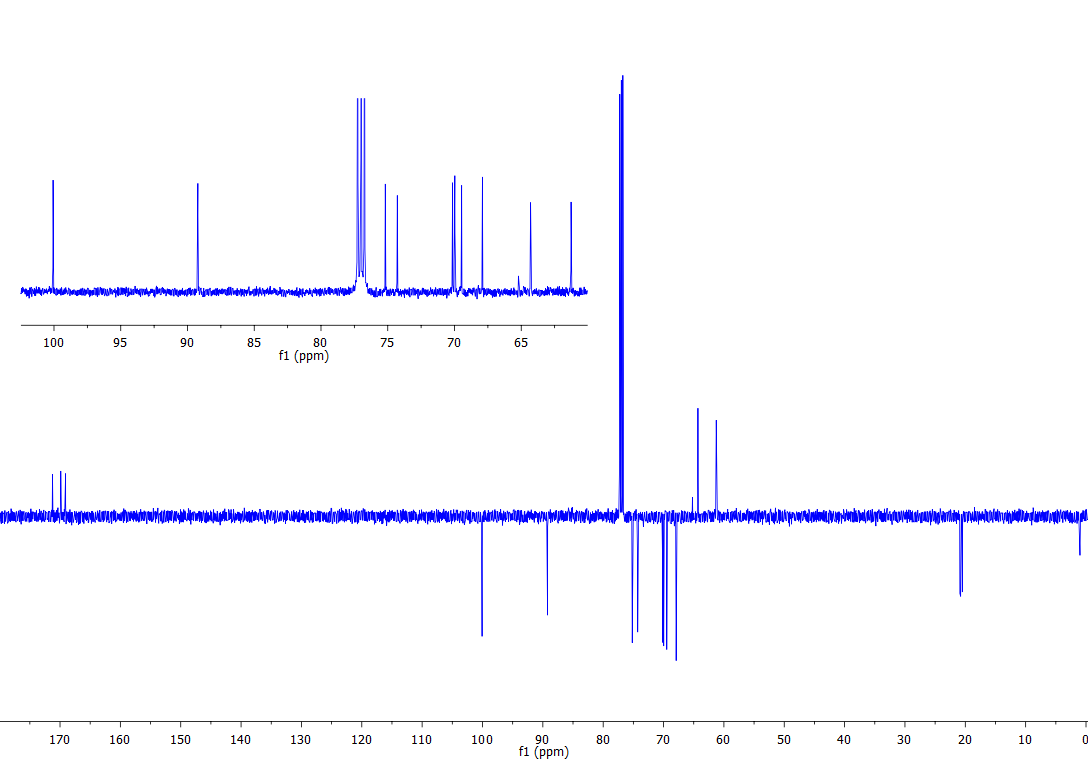


**Figure S9** Jmod and ^13^C NMR spectra (CDCl_3_, 298K) of 2,3-di-*O*-acetyl-β-d-xylopyranosyl-(1,4)-1,2,3-tri-*O*-acetyl-α-d-xylopyranoside (X2Ac5).


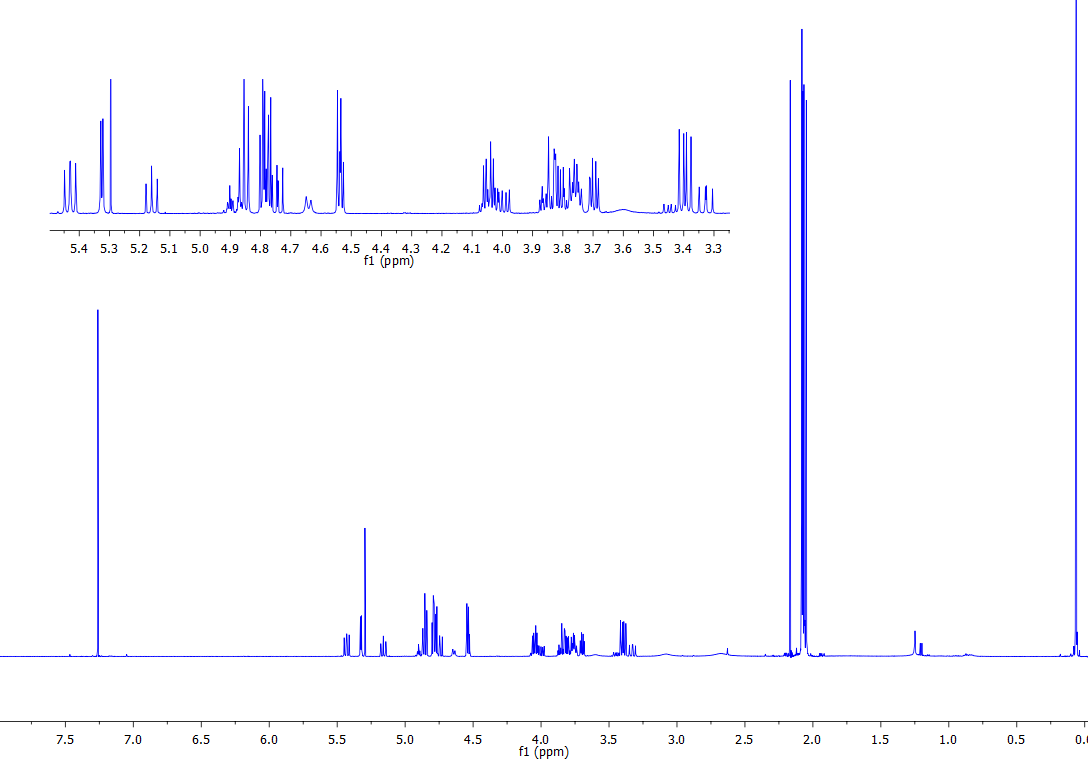


**Figure S10** ^1^H NMR spectrum (CDCl_3_, 298K) of 2,3-di-*O*-acetyl-β-d-xylopyranosyl-(1,4)-2,3-di-*O*-acetyl-d-xylopyranose (X2Ac4).
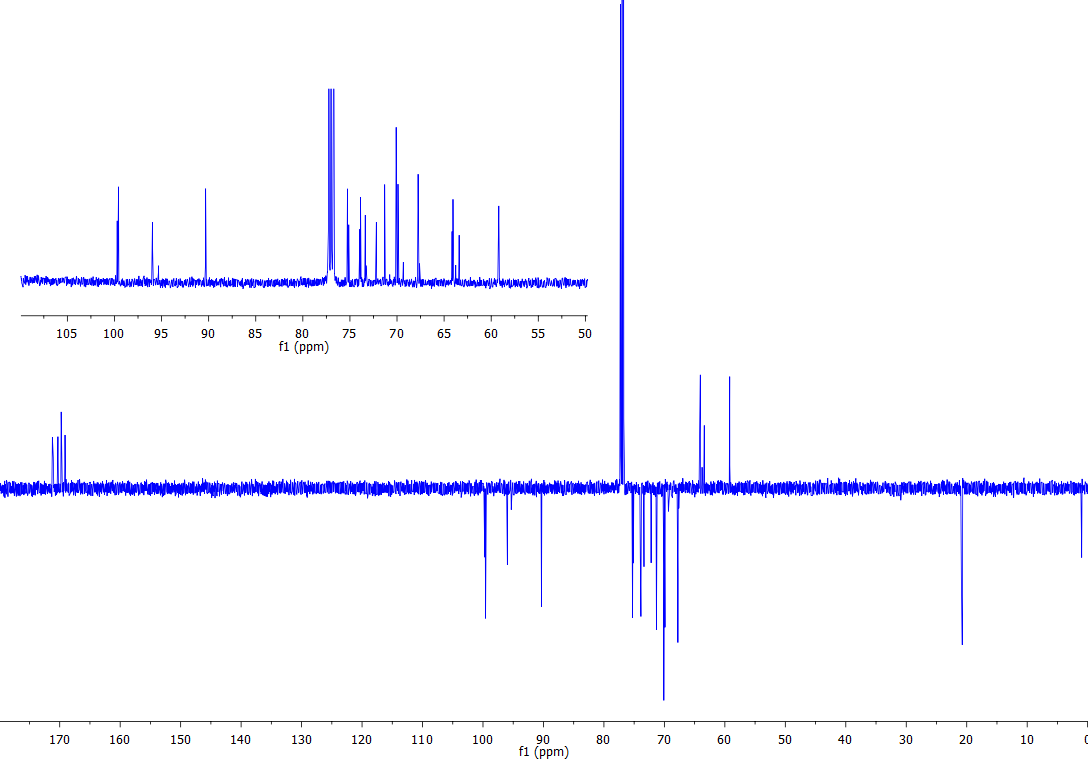


**Figure S11** Jmod and ^13^C NMR spectra (CDCl_3_, 298K) of 2,3-di-*O*-acetyl-β-d-xylopyranosyl-(1,4)-2,3-di-*O*-acetyl-d-xylopyranose (X2Ac4).

### References

1. Gottlieb, H. E. *et al.* NMR chemical shifts of common laboratory solvents as trace impurities. *J. Org. Chem.* **62**, 7512–7515 (1997).

2. Bolam, D. N. *et al.* Synthesis of 2,4-dinitrophenyl glycosides of d-xylobiose and d-mannobiose. *Carbohydr. Res.* **312**, 85–89 (1998).

3. Vršanská, M. *et al.* An alternative approach for the synthesis of fluorogenic substrates of endo-β-(1→4)-xylanases and some applications. *Carbohydr. Res.* **343**, 541–548 (2008).

4. Utille, J. P. & Jeacomine, I. Synthesis of a library of allyl α-l-arabinofuranosyl-α- or β-d-xylopyranosides; route to higher oligomers. *Carbohydr. Res.* **342**, 2649–2656 (2007).
